# The cilia and flagella associated protein CFAP52 orchestrated with CFAP45 is required for sperm motility in mice

**DOI:** 10.1016/j.jbc.2023.104858

**Published:** 2023-05-24

**Authors:** Bingbing Wu, Rachel Li, Shuang Ma, Yanjie Ma, Lijun Fan, Chunxiu Gong, Chao Liu, Ling Sun, Li Yuan

**Affiliations:** 1 Guangzhou Women and Children's Medical Center, Guangzhou Medical University, Guangzhou, China; 2 State Key Laboratory of Stem Cell and Reproductive Biology, Institute of Zoology, Chinese Academy of Sciences, Beijing, China; 3 University of the Chinese Academy of Sciences, Beijing, China; 4 International Division, Beijing Academy, Beijing, China; 5 Department of Endocrinology, Genetics, Metabolism, Beijing Children’s Hospital, Capital Medical University, National Center for Children’s Health, Beijing, China; 6 Savaid Medical School, University of Chinese Academy of Sciences, Beijing, China

**Keywords:** ATP hydrolysis, CFAP52, CFAP45, male infertility, midpiece–principal piece junction, sperm motility

## Abstract

Asthenozoospermia characterized by decreased sperm motility is a major cause of male infertility, but the majority of the etiology remains unknown. Here, we showed that the cilia and flagella associated protein 52 (*Cfap52*) gene was predominantly expressed in testis and its deletion in a *Cfap52* knockout mouse model resulted in decreased sperm motility and male infertility. *Cfap52* knockout also led to the disorganization of the midpiece–principal piece junction of the sperm tail but had no effect on the axoneme ultrastructure in spermatozoa. Furthermore, we found that CFAP52 interacted with the cilia and flagella associated protein 45 (CFAP45) and knockout of *Cfap52* decreased the expression level of CFAP45 in sperm flagellum, which further disrupted the microtubule sliding produced by dynein ATPase. Together, our studies demonstrate that CFAP52 plays an essential role in sperm motility by interacting with CFAP45 in sperm flagellum, providing insights into the potential pathogenesis of the infertility of the human *CFAP52* mutations.

Infertility caused by the male factor affects 25 to 50 million couples worldwide (1, 2), usually due to azoospermia, oligozoospermia, asthenozoospermia, and teratozoospermia (3, 4, 5). Asthenozoospermia, a major cause of male infertility, is diagnosed as total sperm motility under 50% and progressive motility under 32% (6). It has been reported that many factors are associated with asthenozoospermia, including genetic factors, lifestyles, and environmental exposures (6, 7, 8). Among them, most lifestyle- and environmental-related asthenozoospermia can be treated by diet or drugs, but the genetics-related asthenozoospermia still cannot cope with medicines unless treated by intracytoplasmic sperm injection. Nonetheless, the mechanisms underlying most genetics-related asthenozoospermia remain largely unknown.

The movement of spermatozoon is driven by the flagellum. The flagellum makes up about 90% of the length of the sperm whose integrity is essential for sperm motility and fertilization (9, 10). The core structure of sperm flagellum is the axoneme composed of “9 + 2” microtubules, wherein a central microtubule pair is surrounded by 9 peripheral doublet microtubule doublets (11). In doublet microtubule doublets, 10 protofilaments of the B-tubule are connected to 13 protofilaments of the A-tubule at the inner and outer junction (12). Axonemal dyneins bound on A-tubules slide on the neighboring B-tubules, which are powered by ATP hydrolysis, and then this sliding propagates along the axoneme, resulting in a bending force (13). Mutations in several genes have been identified as being associated with asthenozoospermia, including the cilia and flagella associated protein (CFAP) family, such as *CFAP43*, *CFAP44*, *CFAP45*, *CFAP58*, *CFAP65, CFAP69*, and *CFAP251* (3, 14, 15, 16, 17, 18, 19, 20, 21). Despite recent progresses in understanding the pathogenesis of asthenozoospermia, the functional roles of the CFAP family in maintaining sperm motility and flagellar structure have not been fully understood.

Previous studies have shown that CFAP52 (also named WD repeat domain 16 WDR16) is functionally associated with motile cilia, either in the embryonic node or in the ependyma (22, 23). *CFAP52* mutations may cause motile ciliopathy (21, 23). Furthermore, CFAP52 is localized to the proximal region of respiratory cilia and the sperm tail (21, 24). Notably, a homozygous deletion of *CFAP52* exon 2 mutation (c.70 + 1535_270 + 360del, p.His25Argfs∗8) resulted in male infertility. CFAP52 has been recently identified in CFAP45 immunoprecipitates from isolated porcine respiratory cells by proteomic profiling. Deficiency of *CFAP45* in humans and mice has been reported that led to situs inversus totalis and asthenospermia (21). However, the pathogenic mechanism of CFAP52 dysfunction leading to male infertility and whether there is a functional link between CFAP52 and CFAP45 in sperm tail remain unclear. In the present study, we used a *Cfap52* knockout mouse model to investigate the function of CFAP52 in male reproduction. We demonstrated that knockout of *Cfap52* significantly decreased sperm motility and, in turn, rendered male infertility. *Cfap52* knockout also led to disorganization of the midpiece–principal piece junction of the sperm tail. Moreover, we found that CFAP52 interacted with CFAP45 and stabilized CFAP45 in the sperm flagella and the deficiency of *Cfap52* disrupted the microtubule sliding produced by dynein ATPase, thus inhibiting sperm tail bending. Therefore, our studies provide a potential mechanism for asthenozoospermia.

## Results

### *Cfap52* knockout leads to male infertility

**Figure 1:**
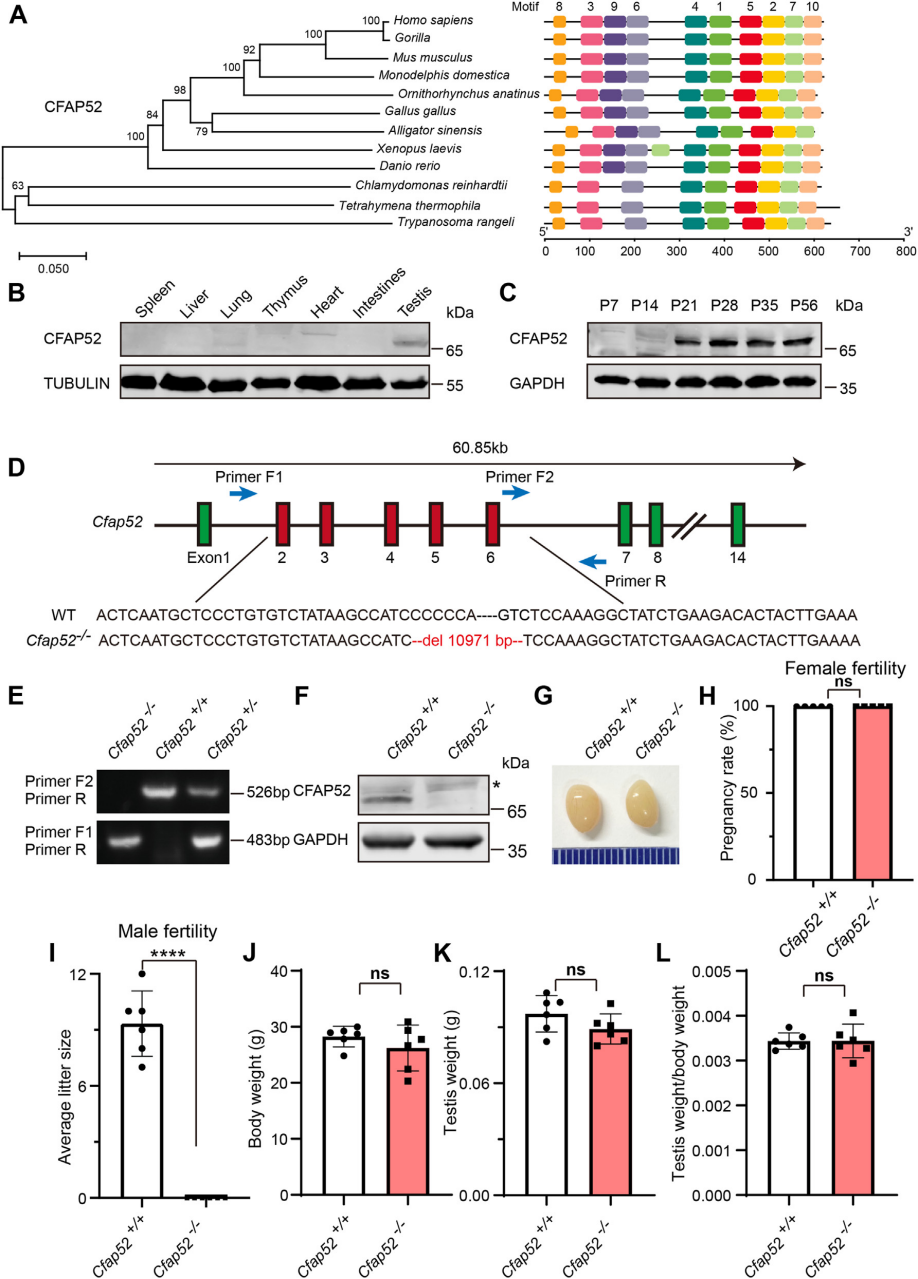
***Cfap52* knockout leads to male infertility.***A*, multiple species phylogenetic tree of CFAP52. Motifs identified in *Mus musculus* CFAP52 and 11 other species. *B*, CFAP52 was predominately expressed in testis. Immunoblotting of CFAP52 was performed in spleen, liver, lung, thymus, heart, intestines, and testis. TUBULIN served as the loading control. *C*, CFAP52 was expressed starting in P14 testes. GAPDH served as the loading control. *D*, generation of *Cfap52* knockout mice lacking exons 2 to 6. *E*, genotyping to identify *Cfap52* knockout mice. *F*, immunoblotting of CFAP52 in *Cfap52*^*+/+*^ and *Cfap52*^*−/−*^ testes. GAPDH served as the control; *asterisks* indicate nonspecific bands. *G*, the size of testes was similar in *Cfap52*^*+/+*^ and *Cfap52*^*−/−*^ mice. *H*, pregnancy rates of *Cfap52*^*+/+*^ and *Cfap52*^*−/−*^ female mice at 2 months (n = 5 independent experiments). No obvious differences were observed between *Cfap52*^*+/+*^ and *Cfap52*^*−/−*^ female mice. *I*, the average litter size of *Cfap52*^*+/+*^ and *Cfap52*^*−/−*^male mice in 2 months (n = 6 independent experiments); *Cfap52*^*−/−*^male mice were completely sterile. Data are presented as mean ± SD. ∗∗∗∗*p* < 0.0001. *J*, body weights of *Cfap52*^*+/+*^ and *Cfap52*^*−/−*^ male mice (n = 6 independent experiments). *K*, testis weights of *Cfap52*^*+/+*^ and *Cfap52*^*−/−*^ male mice (n = 6 independent experiments). *L*, ratio of testis weight to body weight in *Cfap52*^*+/+*^ and *Cfap52*^*−/−*^ male mice (n = 6 independent experiments). Data are presented as mean ± SD. ns: indicates no difference.

CFAP52 is evolutionarily conserved in ciliated organisms (25), and it has been identified as a component of motile cilia in *Trypanosoma brucei* (26). Using the MEME Suite online tool (http://meme-suite.org/tools/meme) (27), we identified 10 motifs in CFAP52 that were conserved in 12 species (Fig. 1*A*). Among them, motifs 1, 2, 3, 4, 5, 6, 7, 8, and 10 were highly conserved across species, whereas motif 9 was only conserved in vertebrates, suggesting that CFAP52 has conserved functions in different species. To study the biological function of CFAP52, we first examined its expression pattern in different tissues and found that it was predominantly expressed in testis and slightly expressed in lung (Fig. 1*B*). Further immunoblotting of mouse testis lysates prepared from different days after birth was carried out. CFAP52 was weakly detected in the testis at postnatal day 14 (P14), and the level increased continuously from postnatal P21 onward (Fig. 1*C*). This time course corresponded with the onset of spermiogenesis, suggesting that CFAP52 might have functional role in spermatozoa.

To determine the physiological role of CFAP52 during spermatogenesis, *Cfap52*^*−/−*^ mice were generated by the CRISPR-Cas9 system to delete exons 2 to 6 of the *Cfap52* gene (Fig. 1*D*). *Cfap52*^*−/−*^ mice were genotyped by genomic DNA sequencing and further confirmed by polymerase chain reaction (PCR), as seen by the 526 bp band for *Cfap52*^*+/+*^ mice, 483 bp band for *Cfap52*^*−/−*^ mice, and two bands of 526 bp and 483 bp for *Cfap52*^*+/−*^ mice (Fig. 1*E*). Immunoblotting analysis validated that CFAP52 was successfully eliminated in the total protein extracts from *Cfap52*^*−/−*^ testes (Fig. 1*F*). Fifty-two percent (14/27) of *Cfap52*^*−/−*^ mice exhibited severe hydrocephalus and died within 2 months. We next examined the fertility of 2-month-old male and female *Cfap52*^*−/−*^ mice. Female *Cfap52*^*−/−*^ mice could generate offspring after mating with WT adult males, similar to the *Cfap52*^*+/+*^ female mice (Fig. 1*H*). Although male *Cfap52*^*−/−*^ mice exhibited normal mounting behaviors and produced coital plugs, they failed to produce any offspring after mating with WT adult female mice (Fig. 1*I*). Taken together, inactivation of *Cfap52* results in male infertility.

### *Cfap52* knockout mice show normal spermatogenesis

To further investigate the cause of male infertility, we first examined *Cfap52*^*−/−*^ testis at gross and histological levels. *Cfap52* knockout did not affect either testis size (Fig. 1*G*) or the ratio of testis weight to body weight (Fig. 1, *J*–*L*). Histological sections stained with hematoxylin and eosin (H&E) revealed that seminiferous tubules of *Cfap52*^*−/−*^ mice displayed normal structure and no obvious defects compared with that of *Cfap52*^*+/+*^ mice (Fig. 2*A*).

**Figure 2:**
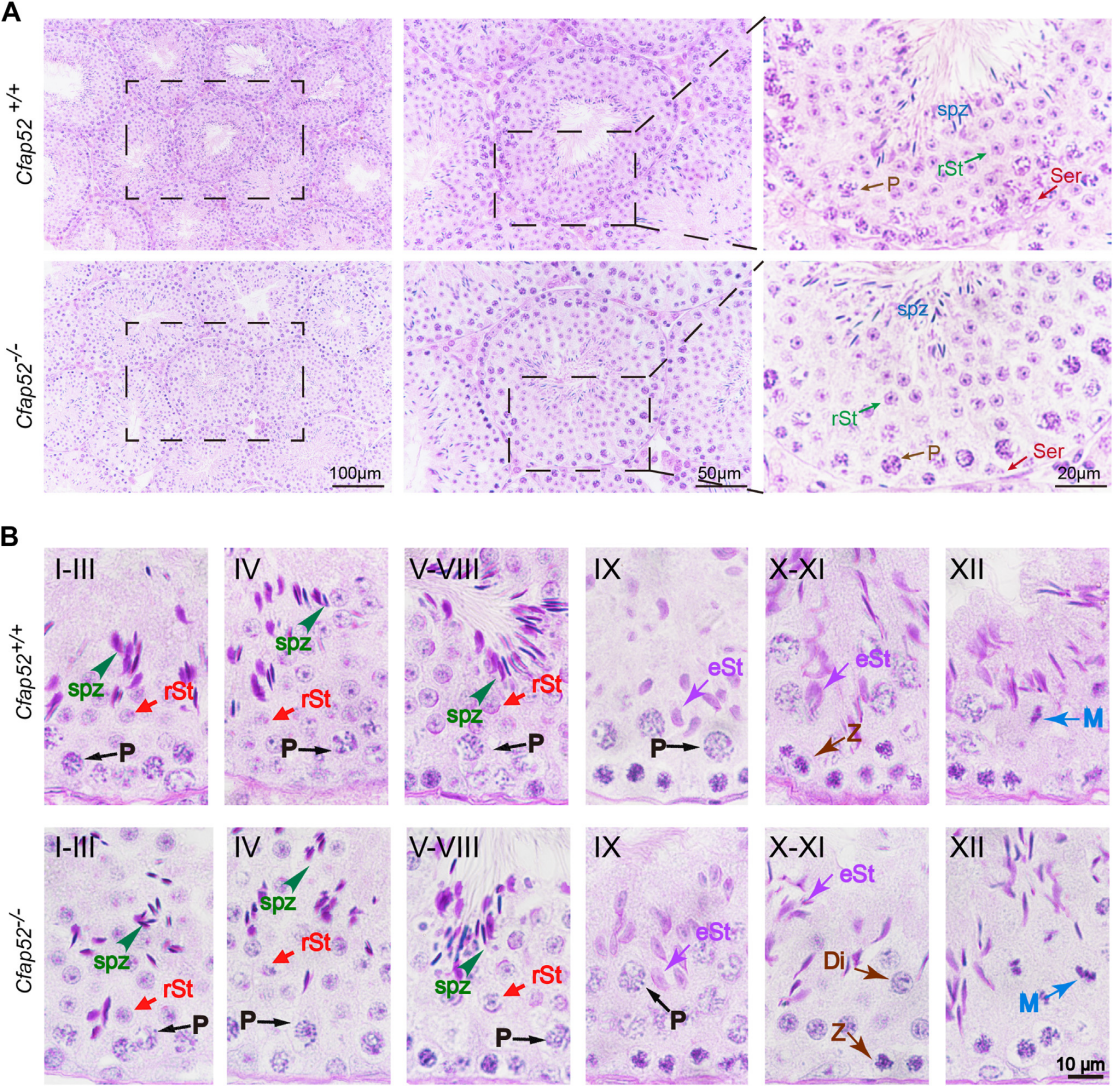
***S*permatogenic process is not affected by *Cfap52* knockout.***A*, histological analysis of the seminiferous tubules of *Cfap52*^*+/+*^ and *Cfap52*^*−/−*^ male mice by H&E staining. *B*, PAS staining of testes sections from *Cfap52*^*+/+*^ and *Cfap52*^*−/−*^ male mice. P, pachytene spermatocyte; Z, zygotene spermatocyte; M, meiotic spermatocyte; Di, diplotene spermatocyte; rSt, round spermatid; eSt, elongating spermatid; spz, spermatozoa; Ser, Sertoli cell.

Spermatogenesis is a continuous, cyclic process in which germ cells undergo a series of developments according to a strict schedule (28). The cycle of the seminiferous epithelium can be subdivided into 12 stages, asserted with periodic acid–Schiff (PAS) staining on testicular sections (29). We thus closely examined the morphology of the seminiferous epithelia in a stage-wise manner. The seminiferous tubules of *Cfap52*^*−/−*^ mice had well-organized architecture and regular arrangement of spermatogenic cells, which were similar to that of the *Cfap52*^*+/+*^ testes (Fig. 2*B*). Thus, these results show that the knockout of *Cfap52* does not affect spermatogenesis.

### *Cfap52* knockout mice produce spermatozoa with low motility

**Figure 3:**
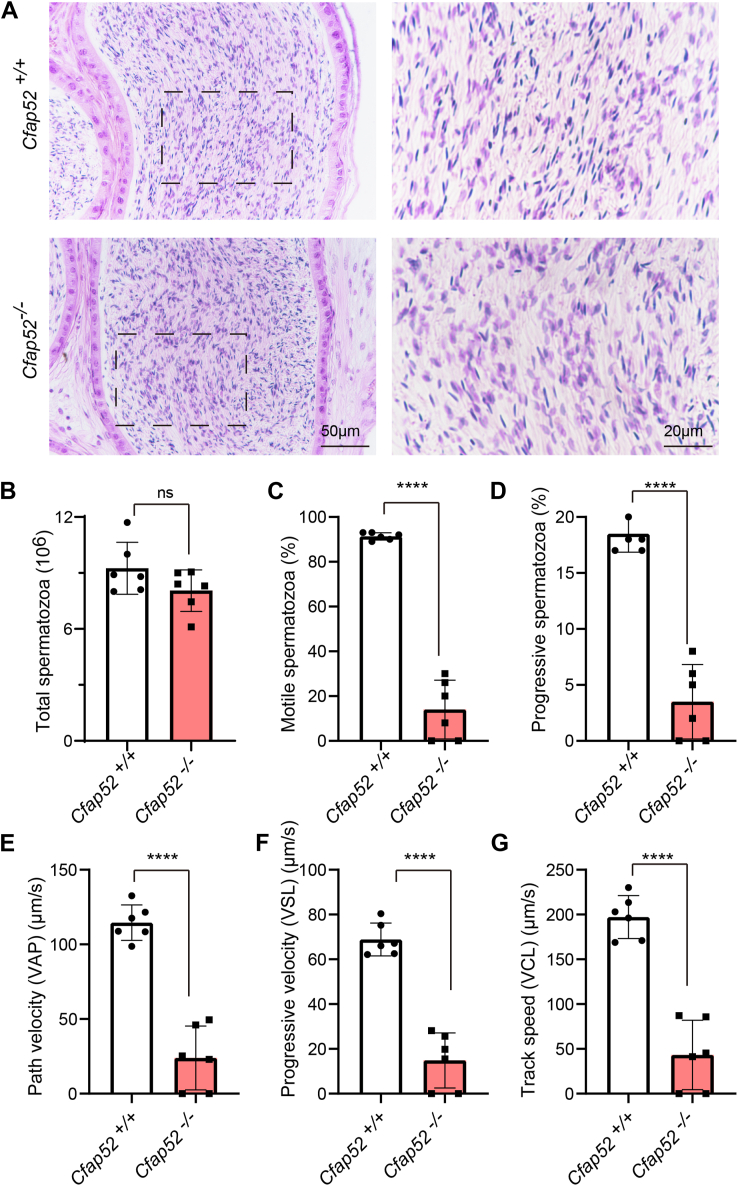
***Cfap52* knockout leads to decreased sperm motility.***A*, H&E staining of the cauda epididymis sections from *Cfap52*^*+/+*^ and *Cfap52*^*−/−*^ mice. *B*, total spermatozoa of the unilateral cauda epididymis in *Cfap52*^*+/+*^ and *Cfap52*^*−/−*^ mice (n = 6 independent experiments). *C*, motile spermatozoa in *Cfap52*^*+/+*^ and *Cfap52*^*−/−*^ mice (n = 6 independent experiments). *D*, progressive spermatozoa in *Cfap52*^*+/+*^ and *Cfap52*^*−/−*^ mice (n = 6 independent experiments). *E*, path velocity (VAP) of spermatozoa from *Cfap52*^*+/+*^ and *Cfap52*^*−/−*^ mice (n = 6 independent experiments). *F*, progressive velocity (VSL) of spermatozoa from *Cfap52*^*+/+*^ and *Cfap52*^*−/−*^ mice (n = 6 independent experiments). *G*, track speed (VCL) of spermatozoa from *Cfap52*^*+/+*^ and *Cfap52*^*−/−*^ mice (n = 6 independent experiments). Data are presented as mean ± SD. ns: indicates no difference. ∗∗∗∗*p* < 0.0001.

We then examined the cross sections of caudal epididymis of *Cfap52*^*−/−*^ mice by H&E staining (Fig. 3*A*) and found no obvious difference between *Cfap52*^*−/−*^ and *Cfap52*^*+/+*^ mice. Sperm count in *Cfap52*^*−/−*^ caudal epididymis was indistinguishable from its wildtype counterpart (Fig. 3*B*). The motility of sperm within the cauda epididymis of *Cfap52*^*−/−*^ and *Cfap52*^*+/+*^ mice was further measured by the computer-assisted semen analysis (CASA) system. As shown in Figure 3, *C* and *D*, *Cfap52*^*+/+*^ mice produced 91.33 ± 1.63% motile sperm including 18.50 ± 1.64% progressive sperm, whereas *Cfap52*^*−/−*^ mice produced only 14.00 ± 13.15% motile sperm including 3.50 ± 3.32% progressive sperm. Furthermore, other motility-related parameters showed significant differences in *Cfap52*^*−/−*^ mice compared with that of *Cfap52*^*+/+*^ mice including the path velocity (VAP), progressive velocity (VSL) and track speed (VCL) (Fig. 3, *E*–*G*). These results indicate that CFAP52 is essential for sperm motility.

### Midpiece and principal piece junction is disorganized in *Cfap52* knockout spermatozoa

**Figure 4:**
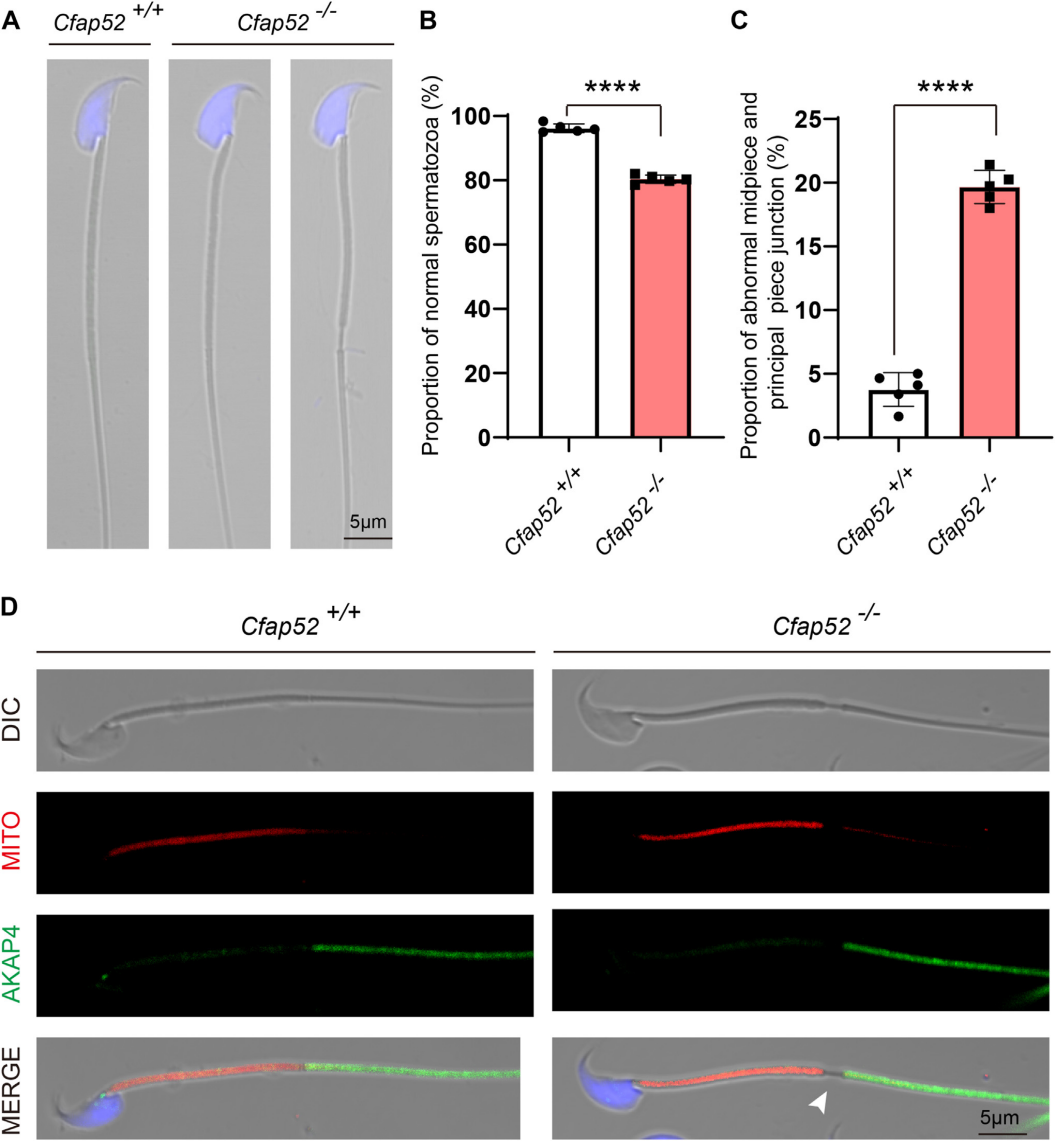
***Cfap52* knockout leads to disorganization of the midpiece–principal piece junction of the sperm tail.***A*, morphology of the cauda epididymal spermatozoa from *Cfap52*^*+/+*^ and *Cfap52*^*−/−*^ mice. *B*, quantification ratio of normal spermatozoa from *Cfap52*^*+/+*^ and *Cfap52*^*−/−*^ mice (n = 5 independent experiments). *C*, quantification ratio of abnormal midpiece and principal piece junction spermatozoa from *Cfap52*^*+/+*^ and *Cfap52*^*−/−*^ mice. At least 200 spermatozoa per mouse of each genotype (n = 5) were counted. Data are presented as mean ± SD. ∗∗∗∗*p* < 0.0001. *D*, fluorescence staining of MitoTracker *Deep Red* and AKAP4 in Cfa*p52*^*+/+*^ and *Cfap52*^*−/−*^ spermatozoa; *white arrowhead* indicates disorganization of the midpiece–principal piece junction of the sperm tail.

The sperm flagellum harbors the specific periaxonemal structures, *i.e.*, a helical mitochondrial sheath in the midpiece, the fibrous sheath in the principal piece, and outer dense fibers in the midpiece and the proximal part of the principal piece, all of which are not found in other motile cilia (14). As shown in Figure 4, *A*–*C*, 19.66% ± 1.30 of spermatozoa isolated from the caudal epididymis of *Cfap52*^*−/−*^ mice displayed a thinning near the annulus that normally connects the midpiece with the principal piece of the flagellum, compared with 3.77% ± 1.32 of this malformation in *Cfap52*^*+/+*^ mice (Fig. 4, *A*–*C*). Immunofluorescence staining with MitoTracker marking the midpiece and an antibody against AKAP4 labeling the principal piece showed that the midpiece and principal piece of the sperm tail was disconnected in the spermatozoa (Fig. 4*D*, arrowhead).

**Figure 5:**
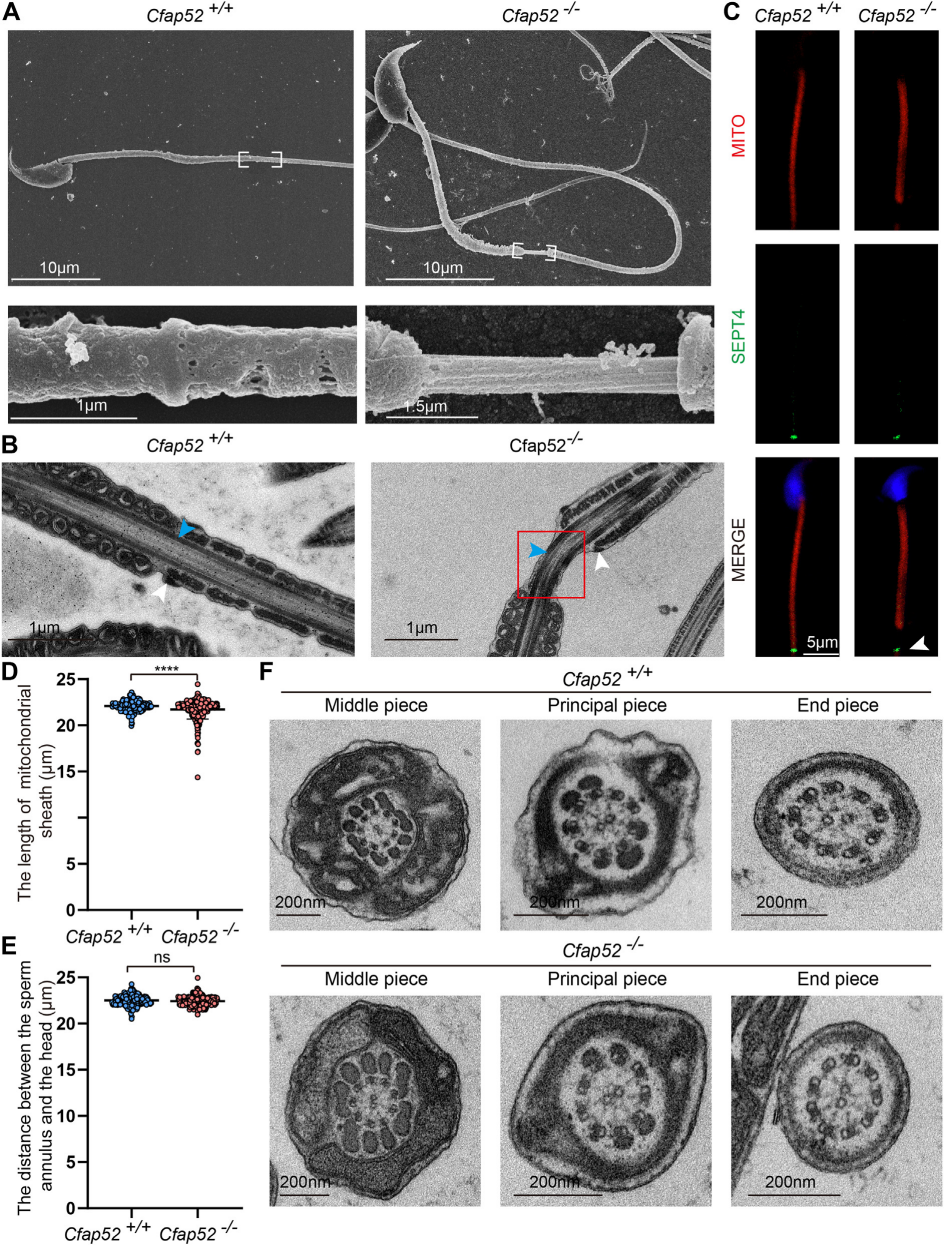
**Ultrastructure of *Cfap52* knockout mice sperm flagella.***A*, scanning electron microscope analysis of spermatozoa from the cauda epididymidis of *Cfap52*^*+/+*^ and *Cfap52*^*−/−*^ mice. Magnified images (*white boxes*) are shown in the *lower panels*. *B*, transmission electron microscopy analysis of spermatozoa from the cauda epididymidis of *Cfap52*^*+/+*^ and *Cfap52*^*−/−*^ mice; *white arrowheads* indicate the annulus region and *blue arrowheads* indicate the outer dense fibers. The *red box* indicates the separation of the midpiece–principal piece junction of the sperm tail. *C*, fluorescence staining of MitoTracker *Deep Red* and SEPT4 in Cfa*p52*^*+/+*^ and *Cfap52*^*−/−*^ spermatozoa; *white arrowhead* indicates the annulus region. *D*, significant differences in the length of *Cfap52*^*+/+*^ and *Cfap52*^*−/−*^ sperm mitochondrial sheath. At least 100 spermatozoa per mouse of each genotype (n = 3) were counted, and a total of at least 300 spermatozoa were counted. Data are presented as mean ± SD. ∗∗∗∗*p* < 0.0001. *E*, the distance between the sperm annulus and the head in *Cfap52*^*+/+*^ and *Cfap52*^*−/−*^ male mice. At least 100 spermatozoa per mouse of each genotype (n = 3) were counted, and a total of at least 300 spermatozoa were counted. Data are presented as mean ± SD. ns: indicates no difference. *F*, cross sections of *Cfap52*^*−/−*^ sperm flagellum revealed normal ultrastructure of axonemal microtubules and tail accessory structures.

Scanning electron microscopy and transmission electron microscopy (TEM) further revealed the thinning region in *Cfap52*^*−/−*^ spermatozoa was axoneme solely enveloped by outer dense fibers (Fig. 5*A*, white box and 5B, blue arrowhead). The sperm annulus is a septin-based structure composed of several septins and essential for the structural and mechanical integrity of mammalian spermatozoa (30, 31, 32). Next, we stained spermatozoa using MitoTracker and SEPT4, respectively, to visualize mitochondrial sheath and annulus and found that the length of the mitochondrial sheath in *Cfap52*^*−/−*^ spermatozoa was shorter than that in *Cfap52*^*+/+*^ spermatozoa (Fig. 5, *C* and *D*). The distance between SEPT4-positive annulus and sperm head in *Cfap52*^*−/−*^ sperm flagella did not differ from that of its wildtype counterpart (Fig. 5, *C*, arrowhead, and *E*). Moreover, TEM cross sections of *Cfap52*^*−/−*^ sperm flagellar axonemes showed normal 9 + 2 microtubular organization and tail accessory structures (Fig. 5*F*).

In summary, *Cfap52*^*−/−*^ male mice displayed asthenozoospermia with disconnected midpiece and principal piece albeit without detectable ultrastructural defects in organization or assembly of the axoneme.

### CFAP52 deficiency destabilizes CFAP45 in sperm flagella

Previous studies have shown that CFAP52 located to the sperm flagellum, suggesting that CFAP52 might have an important impact on the stability, maintenance, or motility of the sperm flagella (24). In *Chlamydomonas*, flagellar-associated proteins 52 (FAP52) and flagellar-associated proteins 45 (FAP45) have been identified as microtubule inner proteins and played an essential role in axoneme stability (12). Moreover, the double mutant *fap45fap52* swam with a significantly lower beat compared with that of the wildtype. Deficiency of *CFAP45* in humans and mice is associated with asthenozoospermia (21). Thus, it is possible that CFAP52 might interact with CFAP45 to regulate sperm flagellar beating in mice and humans.

**Figure 6:**
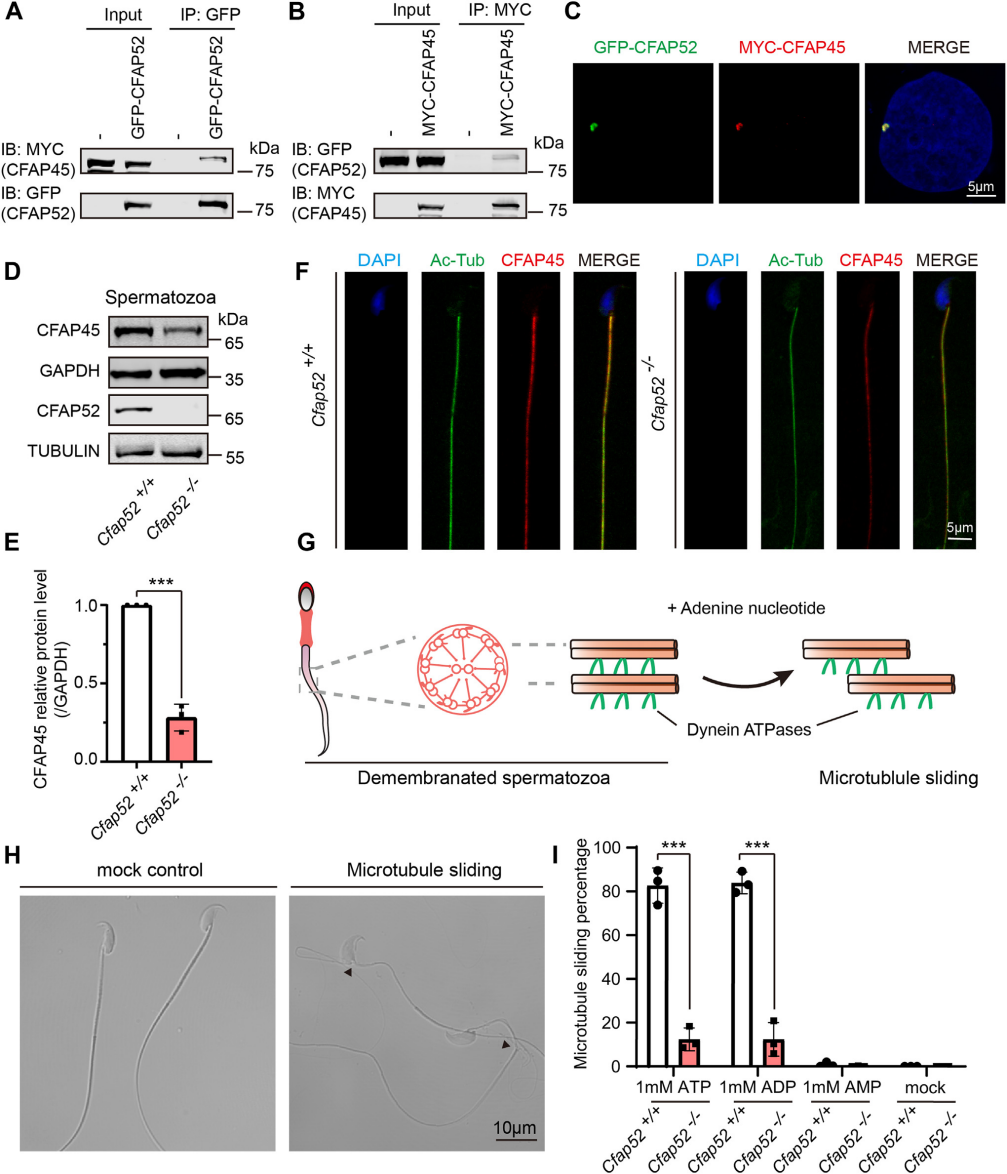
**CFAP52 interacts with CFAP45 to promote microtubule sliding.***A* and *B*, CFAP52 interacts with CFAP45. pCS2-MYC-CFAP45 and pEGFP-GFP-CFAP52 plasmids were cotransfected into HEK293T cells. Forty-eight hours after transfection, the cell lysates were collected for immunoprecipitation with anti-GFP antibody or anti-MYC antibodies and analyzed with anti-MYC or anti-GFP antibodies. IB, immunoblotting; IP, immunoprecipitation. *C*, CFAP52 colocalized with CFAP45 in HeLa cells. pCS2-MYC-CFAP45 and pEGFP-GFP-CFAP52 plasmids were cotransfected into HeLa cells. Twenty-four hours after transfection, the cells were fixed and stained with anti-MYC and anti-GFP antibodies, and the nuclei were stained with DAPI (*blue*). *D*, Western blotting analysis to show CFAP45 protein levels in spermatozoa released from the caudal epididymis of *Cfap52*^*+/+*^ and *Cfap52*^*−/−*^ mice. GAPDH served as the loading control. Immunoblotting of CFAP52 in spermatozoa released from the caudal epididymis of *Cfap52*^*+/+*^ and *Cfap52*^*−/−*^ mice. TUBULIN served as the loading control. *E*, quantification of the CFAP45 relative protein levels using the Odyssey software and compared with the control group (n = 3 independent experiments). Data are presented as mean ± SD. ∗∗∗*p* < 0.001. *F*, immunofluorescence of AcTub (*green*) and CFAP45 (red) in spermatozoa released from the caudal epididymis of *Cfap52*^*+/+*^ and *Cfap52*^*−/−*^ mice. Nuclei were stained with DAPI (*blue*). *G*, schematic diagram for microtubule sliding assay to reconstitute dynein ATPase activity. *H*, microtubule sliding assay to reconstitute dynein ATPase activity. Microtubule sliding was observed (*black arrows*). *I*, ATP, 1 mM, or ADP, 1 mM, alone did not significantly reactivate sliding percentage of *Cfap52*^*−/−*^ spermatozoa compared with the control group. At least 200 spermatozoa per mouse of each genotype (n = 3) were counted by light microscopy. Data are presented as mean ± SD. ∗∗∗*p* < 0.001. AMP, 1 mM, alone showed negligible activation, which was similar to the control.

To study the relationship between CFAP52 and CFAP45, reciprocal coimmunoprecipitation assays were then carried out. Epitope-tagged CFAP52 and CFAP45 expressed in HEK293T cells were able to interact with the other in a reciprocal manner (Fig. 6, *A* and *B*). Furthermore, immunostaining detected an extensive overlap between GFP-CFAP52 and MYC-CFAP45 in HeLa cells (Fig. 6*C*), suggesting that CFAP52 colocalizes with CFAP45 on sperm tail. We next analyzed CFAP52 and CFAP45 protein expression levels in spermatozoa released from the caudal epididymis of *Cfap52*^*+/+*^ and *Cfap52*^*−/−*^. CFAP52 was detectable in *Cfap52*^*+/+*^ but not *Cfap52*^*−/−*^ sperm lysates (Fig. 6*D*). Surprisingly, CFAP45 protein expression level from the *Cfap52*^*−/−*^ sperm lysate was reduced to 28% of the control group (Fig. 6, *D* and *E*), which promoted us to examine the flagellar localization of CFAP45 in *Cfap52*^*−/−*^ spermatozoa. The results showed that the CFAP45 signal retained on sperm flagella of *Cfap52*^*−/−*^ spermatozoa but clearly decreased compared with that of the *Cfap52*^*+/+*^ spermatozoa (Fig. 6*F*). These results suggest that deficiency of *Cfap52* destabilizes the CFAP45 protein in sperm flagella.

### Dynein-driven microtubule sliding is disrupted in *Cfap52* knockout spermatozoa

Flagellar motility depending on adenosine triphosphate (ATP) hydrolysis is generated by the sliding of adjacent doublet microtubules *via* the activity of dynein arm ATPases (33, 34). Thus, adenine nucleotides including ATP, ADP, AMP, and cAMP are required for sperm motility (35, 36, 37, 38, 39). To understand why loss of *Cfap52* leads to low sperm motility, we investigated microtubule sliding using a demembranated spermatozoa model (Fig. 6*G*) (40). When the demembranated spermatozoa were treated with ATP or ADP, the microtubule extruded mainly from the neck or the annulus region of the flagella (Fig. 6*H*, black arrowheads) but not in the untreated (mock) group (Fig. 6*H*). We next evaluated dynein ATPase activation *via* microtubule sliding of *Cfap52*^*−/−*^ mouse spermatozoa using ATP, ADP, and AMP. AMP, 1 mM, alone failed to reactivate microtubule sliding in both *Cfap52*^*+/+*^ and *Cfap52*^*−/−*^ spermatozoa, similar to the mock group (Fig. 6*I*). Addition of either 1 mM ATP or 1 mM ADP significantly reactivated microtubule sliding of *Cfap52*^*+/+*^ but not *Cfap52*^*−/−*^ spermatozoa. These results suggest that decreased sperm motility of *Cfap52*^*−/−*^ mice results from the defect in activation of the microtubule sliding produced by dynein ATPase.

## Discussion

CFAP52 is conserved across ciliated species (25), and it has been identified as a component of flagella in *Chlamydomonas reinhardtii* (41). Knockdown of *CFAP52* in zebrafish led to ciliary phenotype of hydrocephalus, and human mutations were reported to be associated with *situs inversus totalis* and male infertility (22, 23). Consistent with these previous results, we found that the knockout of *Cfap52* also resulted in hydrocephalus (data not shown). Most importantly, we found that *Cfap52*^*−/−*^ male mice were completely sterile due to decreased sperm motility. Abnormalities of the axoneme and periaxonemal structures mainly result in asthenozoospermia (42, 43, 44, 45), which are associated with morphological flagellar defects such as irregular 9 + 2 axoneme structure, missing outer dense fibers, abnormal mitochondrial sheath, and disorganized fibrous sheath (11, 46, 47, 48). Here, the axoneme ultrastructure of *Cfap52*^*−/−*^ spermatozoa was unaltered (Fig. 5*D*). The knockout of *Cfap52* did not disrupt the formation of the annulus but disconnected the midpiece from the principal piece (Fig. 5, *A*–*C*). This defect may result from mechanical stress during sperm transit through the epididymis. Because of only around 20% spermatozoa with this defect, it should not be the major factor that resulted in male infertility (Fig. 3, *C*–*G*).

FAP52 and FAP45, the homologues of CFAP52 and CFAP45 in *Chlamydomonas*, have been reported to bind to the inside of microtubules and stabilize ciliary axonemes (12). Recently, it has been reported that lack of *CFAP45* in both humans and mice resulted in asthenozoospermia (21). As a CFAP45-binding partner, CFAP52 may play a role similar to that of CFAP45 because *Cfap52* knockout also leads to asthenozoospermia-like phenotype in mice and the expression level of CFAP45 was significantly decreased in *Cfap52*^*−/−*^ sperm lysate (Fig. 6, *D* and *E*). Moreover, CFAP45 signal intensity on *Cfap52*^*−/−*^ sperm flagella reduced dramatically (Fig. 6*F*). Thus, CFAP52 and CFAP45 may work as a complex to mutually stabilize themselves and cooperatively regulate sperm motility.

**Figure 7:**
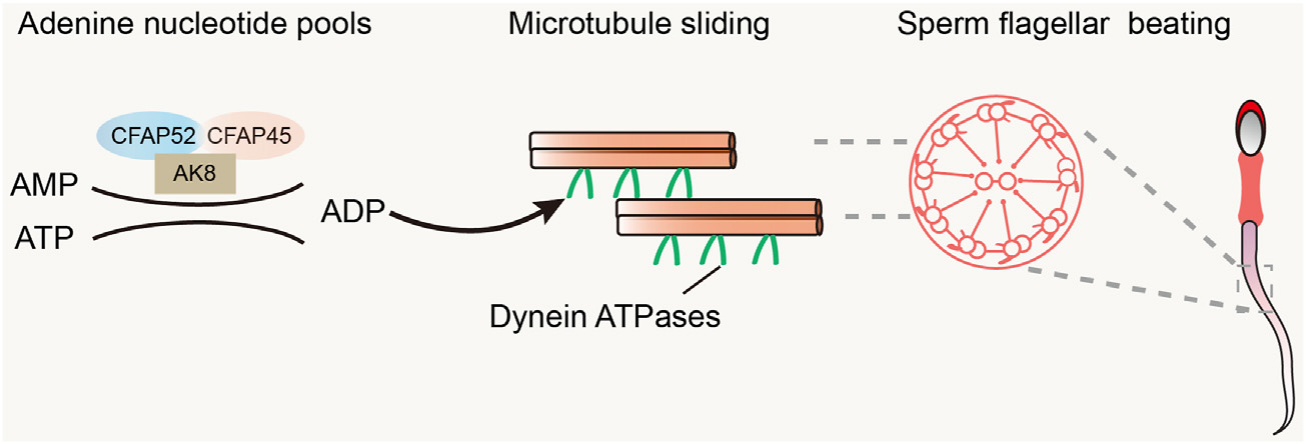
**Proposed model for the functional role of CFAP52 in sperm motility.** CFAP52–CFAP45 interaction, together with AK8, involved in adenine nucleotide homeostasis to mediate adenine nucleotides transportation to dynein ATPases, in turn promote flagellar beating.

Flagellar motility requires ATP hydrolysis, which converts the energy of ATP into mechanical force by dynein motors (33). It has been reported that CFAP52 and CFAP45 might have a functional cooperation with the dynein ATPase outer dynein arm-associated DNAH11 (21). Adenylate kinase (AK) is an evolutionarily conserved enzyme, which catalyzes the reversible reaction ATP + AMP ↔ 2 ADP and maintains balanced nucleotide pools to support ciliary beating (49, 50). AK8 localizes to mouse sperm flagella (39) and can capture both CFAP45 and CFAP52 in HEK293 cell lysates (21). In addition, CFAP45 can bind AMP and transfer it to AK8 and, in turn, mediates adenine nucleotide homeostasis to ensure that dynein ATPases maintain normal force production through interaction with axonemal microtubule doublets (21). Neither ATP nor ADP alone could reactivate microtubule sliding produced by dynein ATPase in *Cfap52*^*−/−*^ spermatozoa (Fig. 6*I*), which is similar to that in *Cfap45*^*−/−*^ spermatozoa. Based on these results, we speculate that CFAP52–CFAP45 interaction, together with AK8, is involved in adenine nucleotide homeostasis to mediate adenine nucleotides transportation to dynein ATPases, in turn promoting flagellar beating (Fig. 7).

In conclusion, we show that *Cfap52* is essential for male fertility, and knockout of this gene leads to asthenozoospermia-like phenotype in mice. CFAP52 interacts with CFAP45, and its deletion results in reduction of CFAP45 protein by 72%, which further disrupts the microtubule sliding produced by dynein ATPase. These findings can provide insights into the pathogenesis of infertility in *CFAP52* human mutations.

## Experimental procedures

### Animals

The mouse *Cfap52* gene (Transcript: ENSMUSG00000020904) is 60.85 kb and contains 14 exons and is located on chromosome 11. Exon 2 to exon 6 was chosen as the target site; *Cfap52* knockout founder mice were generated using the CRISPR-Cas9 system from Cyagen Biosciences. The gRNA and Cas9 mRNA were coinjected into fertilized eggs of C57BL/6J mice to generate heterozygous mutated mice with a 10,971-bp base deletion. The resulting heterozygotes were interbred to obtain offspring, which further were genotyped by genomic DNA sequencing to obtain *Cfap52*^*+/+*^ and *Cfap52*^*−/−*^mice.

For *Cfap52*^*−/−*^ mice, the specific primers were

Forward: 5′-TGAAGCCAGCATTCTGAAGTAG-3′

Reverse: 5′-TTAACCAACGGCCCTGAAATGAT-3′, yielding a 483-bp fragment.

For *Cfap52*^*+/+*^ mice, the specific primers were

Forward: 5′-GCACATCTACTTTCCCACTTAACA-3′

Reverse: 5′-TTAACCAACGGCCCTGAAATGAT-3′, yielding a 526-bp fragment.

All of the animal experiments were performed according to approved institutional animal care and use committee (IACUC) protocols (#08-133) of the Institute of Zoology, Chinese Academy of Sciences.

### Plasmids

Mouse *Cfap52* was obtained from mouse testis cDNA and cloned into the pEGFP-C1 vector using the Clon Express Ultra One Step Cloning Kit (C115, Vazyme). Mouse *Cfap45* was obtained from mouse testis cDNA and cloned into the pCMV-Myc vector using the Clon Express Ultra One Step Cloning Kit (C115, Vazyme).

### Antibodies

Mouse anti-CFAP52 polyclonal antibody (aa 421–620) was generated by Quan Biotech and was used at a 1:500 dilution for Western blotting. Rabbit anti-CFAP45 antibody (HPA043618, Atlas Antibodies) was used at a 1:500 dilution for Western blotting and a 1:400 dilution for immunofluorescence. Mouse anti-GAPDH antibody (AC002, ABclonal) was used at a 1:5000 dilution for Western blotting. Rabbit anti-Tubulin antibody (A6830, ABclonal) was used at a 1:5000 dilution for Western blotting. Mouse anti-GFP antibody (M20004, Abmart) was used at a 1:2000 dilution for Western blotting. Rabbit anti-MYC antibody (BE2011, EASYBIO) was used at a 1:2000 dilution for Western blotting. Rabbit anti-AKAP4 antibody (A14813, ABclonal) was used at a 1:100 dilution for immunofluorescence. Mouse anti-acetylated α tubulin antibody (T7451, Sigma-Aldrich) was used at a 1:200 dilution for immunofluorescence. The secondary antibodies were goat anti-rabbit FITC (1:200, ZF-0311, Zhong Shan Jin Qiao), goat anti-mouse FITC (1:200, ZF-0312, Zhong Shan Jin Qiao), and goat anti-rabbit TRITC (1:200, ZF0313, Zhong Shan Jin Qiao). MitoTracker Deep Red 633 (1:1500 dilution, M22426, Thermo Fisher Scientific) was used for immunofluorescence.

### Immunoprecipitation

Transfected HEK293T cells were lysed in lysis buffer (50 mM Hepes, 250 mM NaCl, 0.1% NP-40, 1 mM PMSF) for 30 min at 4 °C and centrifuged at 12,000×*g* for 15 min. For immunoprecipitation, cell lysates were incubated with antibody about 12 h at 4 °C and then incubated with protein A-Sepharose (GE, 17-1279-03) for 3 h at 4 °C. The precipitants were washed 4 times with lysis buffer, and the immune complexes were eluted with SDS loading buffer containing 2% SDS with 1 M DTT for 10 min at 95 °C and analyzed by immunoblotting.

### Phylogenetic analysis and conserved motifs analysis

Amino acid sequences of CFAP52 of 12 species were downloaded from NCBI (https://www.ncbi.nlm.nih.gov/protein) and UniProt (http://www.uniprot.org). The phylogenetic trees were constructed using MEGA 10.0 (51) with the Neighbor-Joining method (52). Bootstrap analyses were carried out using 1000 replications with the p-distance model (53). Conserved motifs were predicted by MEME (Multiple EM for Motif Elicitation) motif discovery tool (http://meme-suite. org/tools/meme) (27). The minimum and maximum width of each motif was set to be 30 aa and 100 aa, respectively. Each motif must be recognized in at least two sequences. The visual representations of motifs were generated by TBtools (54).

### Immunoblotting

Proteins obtained from lysates were separated by SDS-PAGE and electrotransferred onto a nitrocellulose membrane. The membrane was blocked in 5% skim milk (BD, 232100) and then incubated with corresponding primary antibodies and detected by Alexa Fluor 680- or 800-conjugated goat anti-mouse or Alexa Fluor 680- or 800-conjugated goat anti-rabbit secondary antibodies. Finally, they were scanned using the ODYSSEY Sa Infrared Imaging System (LI-COR Biosciences, RRID:SCR_014579).

### Tissue collection and histological analysis

The testes and caudal epididymis from 2-month-old *Cfap52*^*+/+*^ and *Cfap52*^*−/−*^ mice (n = 6 independent experiments) were dissected immediately after euthanasia. All samples were immediately fixed in 4% (mass/vol) paraformaldehyde for up to 16 h, dehydrated in 70% (vol/vol) ethanol, and embedded in paraffin. For histological analysis, 5-μm sections were mounted on glass slides and stained with H&E. For PAS staining, testes were fixed with Bouin’s fixatives. Slides were stained with PAS and H&E after deparaffinization, and the stages of the seminiferous epithelium cycle and spermatid development were determined.

### Sperm motility assessment using CASA

Spermatozoa were squeezed out from the cauda epididymis of 2-month-old *Cfap52*^*+/+*^ and *Cfap52*^*−/−*^ mice (n = 6 independent experiments) and released in 1 ml phosphate buffered saline (PBS) for 15 min at 37 °C. After incubating, 10 μl sperm liquid taken from each sample was used for the analysis of sperm motility with an Olympus BX51 microscope through a 20× phase objective (OLYMPUS). Viewing areas in each chamber were imaged using a CCD camera (Olympus). The samples were analyzed *via* CASA using the Minitube Sperm Vision Digital Semen Evaluation System (12500/1300, Minitube Group). *Via* CASA, the sperm samples were observed to evaluate the parameters of total sperm motility, including motile spermatozoa, progressive spermatozoa, average path velocity (VAP), progressive velocity (VSL), the track speed (VCL).

### Immunofluorescence

Spermatozoa were released from the cauda epididymis of 2-month-old *Cfap52*^*+/+*^ and *Cfap52*^*−/−*^ mice (n = 3 independent experiments) in PBS at 37 °C for 15 min, then were spread on glass slides for morphological observation or immunostaining. After air drying, spermatozoa were fixed in 4% PFA for 5 min at room temperature, and slides were washed with PBS 3 times and blocked with 5% bovine serum albumin for 30 min at room temperature. The primary antibodies were added to the sections and incubated at 4 °C overnight, followed by incubation with the secondary antibody. The nuclei were stained with DAPI, and images were taken using an LSM 880 microscope (Zeiss) or SP8 microscope (Leica).

### Transmission electron microscopy

The cauda epididymis from 2-month-old *Cfap52*^*+/+*^ and *Cfap52*^*−/−*^ mice (n = 3 independent experiments) were dissected and prefixed in 2.5% (vol/vol) glutaraldehyde in 0.1 M cacodylate buffer at 4 °C overnight. After washing in 0.1 M cacodylate buffer, samples were cut into small pieces of approximately 1 mm^3^, then immersed in 1% OsO_4_ for 1 h at 4 °C. Samples were dehydrated through a graded acetone series (50%, 60%, 70%, 80%, 90%, 100%) and embedded in resin for staining. Ultrathin sections of 60 nm were cut and stained with uranyl acetate and lead citrate. Images were acquired and analyzed using a JEM-1400 transmission electron microscope.

### Scanning electron microscopy

Spermatozoa were released from the cauda epididymis of 2-month-old *Cfap52*^*+/+*^ and *Cfap52*^*−/−*^ mice (n = 3 independent experiments) in PBS at 37 °C for 15 min, centrifuged for 5 min at 500*g*, washed 3 times with phosphate buffer (NaH2PO4 and Na2HPO4·12H2O) and fixed in 2.5% glutaraldehyde solution overnight. Samples were finally dehydrated in graded ethanol, subjected to drying, and coated with gold. Images were acquired and analyzed using a SU8010 scanning electron microscope (SU8010, JEOL).

### Microtubule sliding assay

Microtubule sliding assay was conducted using methods previously reported, with some modifications (55, 56). Spermatozoa were squeezed out from caudal epididymis and released in 50 μl HTF (Sigma-Aldrich, MR-070-D) for 15 min at 37 °C. A 5-μl sample from this suspension was transferred to 100 μl demembranation solution (1 mM EDTA, 50 mM Hepes, 0.2% Triton X-100, pH 7.9), stirred gently for 30 s at 37 °C, and 10 μl of the suspension was transferred to 100 μl reactivation solution (1 mM EDTA, 1 mM adenine nucleotide, 5 mM MgSO4, 33 mM DTT, 50 mM Hepes, pH 7.9) and incubated for 10 min at 37 °C. A 10-μl sample was placed on a glass slide with a coverslip, and sperm with or without sliding were counted.

### Statistical analysis

All of the experiments were repeated at least 3 times, and the results are presented as the mean ± SD. The statistical significance of the differences between the mean values for the different genotypes was measured by the Student’s *t* test with a paired, two-tailed distribution. The data were considered significant for *p* < 0.05.

## Data availability

All data are contained within the article.

## Supporting information

This article contains supporting information.

## Conflict of interest

The authors declare no conflicts of interest with the contents of this article.

## Figures and Tables

**Figure 2A and 3A:**
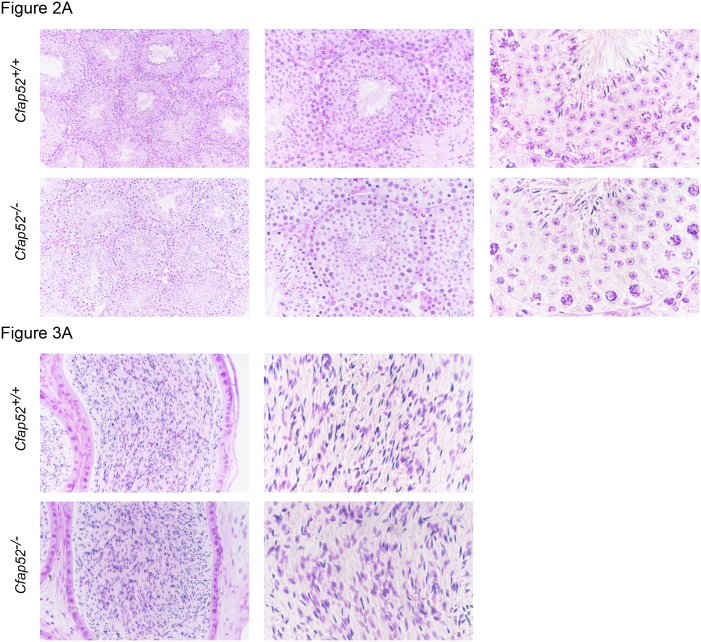
Figure S2A. Histological analysis of the seminiferous tubules of *Cfap52^+/+^* and *Cfap52^–/–^* male mice by H&E staining. Figure S3A. H&E staining of the cauda epididymis sections from *Cfap52^+/+^* and *Cfap52^–/–^* mice.

**Figure 2B:**
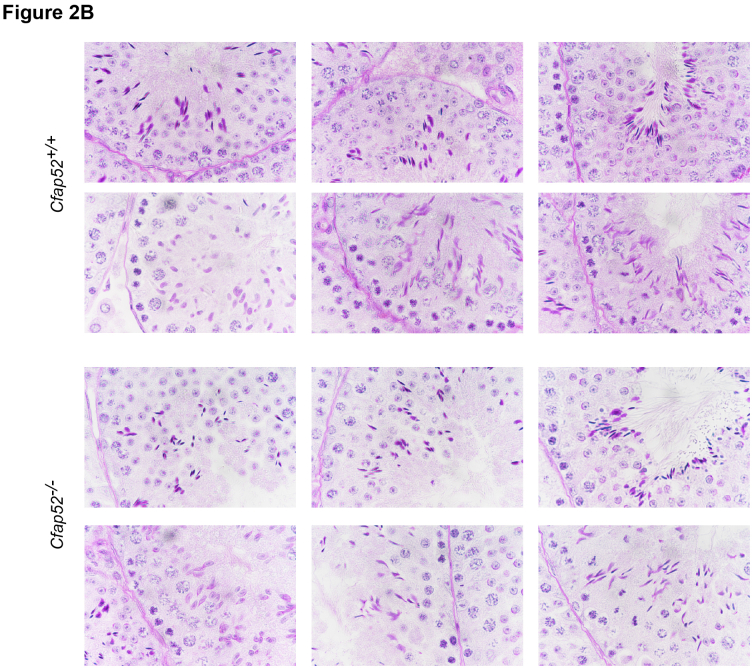
Figure S2B. PAS staining of testes sections from *Cfap52^+/+^* and *Cfap52^–/–^* male mice.

## Supporting information

Figure+S1+legend

Figure S4A Morphology of the cauda epididymal spermatozoa from *Cfap52^+/+^* and *Cfap52^–/–^* mice.

Figure S4D Fluorescence staining of *MitoTracker* Deep Red and AKAP4 in Cfa*p52^+/+^* and *Cfap52^–/–^* spermatozoa; *white arrowhead* indicates disorganization of the midpiece-principal piece junction of the sperm tail.

Figure S5A Scanning electron microscope (SEM) analysis of spermatozoa from the cauda epididymidis of *Cfap52^+/+^* and *Cfap52^-/-^* mice. Magnified images (*white boxes*) are shown in the *lower panels*.

Figure S5B Transmission electron microscopy (TEM) analysis of spermatozoa from the cauda epididymidis of *Cfap52^+/+^* and *Cfap52^-/-^* mice; *white arrowheads* indicate the annulus region and *blue arrowheads* indicate the outer dense fibers. *Red box* indicates the separation of the midpiece-principal piece junction of the sperm tail.

Figure S5C Fluorescence staining of *MitoTracker* Deep Red and SEPT4 in Cfa*p52^+/+^* and *Cfap52^–/–^* spermatozoa; *white arrowhead* indicates the annulus region.

Figure S6C CFAP52 colocalized with CFAP45 in HeLa cells. pCS2-MYC-CFAP45 and pEGFP-GFP-CFAP52 plasmids were cotransfected into HeLa cells. Twenty four hours after transfection, the cells were fixed and stained with anti-MYC and anti-GFP antibodies, and the nuclei were stained with DAPI (*blue*).

Figure S6F Immunofluorescence of AcTub (*green*) and CFAP45 (*red*) in spermatozoa released from the caudal epididymis of *Cfap52^+/+^* and *Cfap52^–/–^* mice. Nuclei were stained with DAPI (*blue*).

Figure S6H Microtubule sliding assay to reconstitute dynein ATPase activity. Microtubule sliding was observed (*black arrows*).
