# Supplementary material for: The cilia and flagella associated protein CFAP52 orchestrated with CFAP45 is required for sperm motility in mice
Source: J Biol Chem. 2023 May 24;299(7):104858. doi: 10.1016/j.jbc.2023.104858 (PMC10319328; doi:10.1016/j.jbc.2023.104858)
Supplement: Figure+S1+legend [file mmc1.docx]

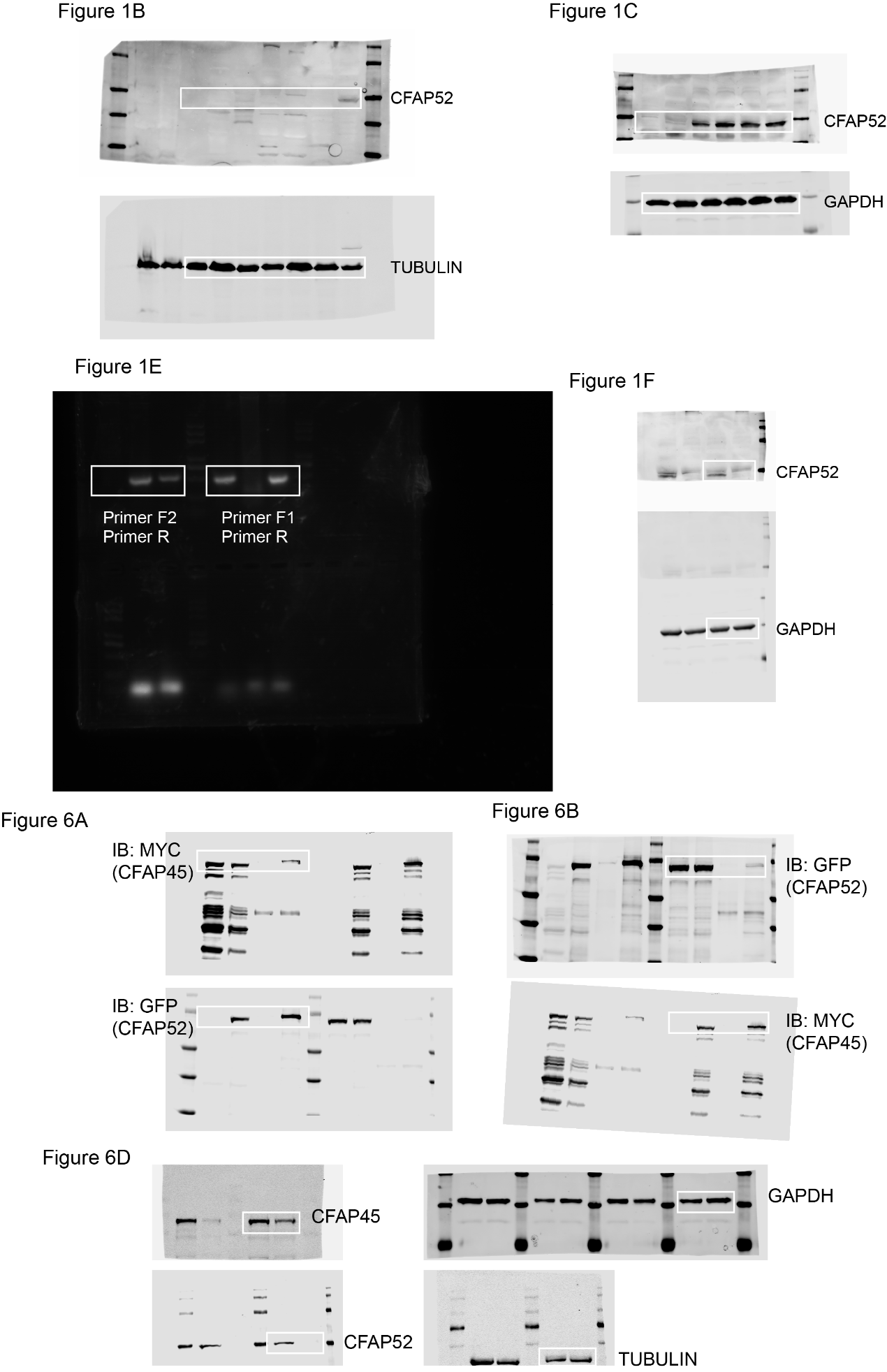


Figure S1 legend is provided below:

Figure S1 (B, C, E, F) CFAP52 was predominately expressed in testis.

Figure S6 (A, B, D) CFAP52 interacted with CFAP45.
