# Supplementary figures and images for: The cilia and flagella associated protein CFAP52 orchestrated with CFAP45 is required for sperm motility in mice
Source: J Biol Chem. 2023 May 24;299(7):104858. doi: 10.1016/j.jbc.2023.104858 (PMC10319328; doi:10.1016/j.jbc.2023.104858)

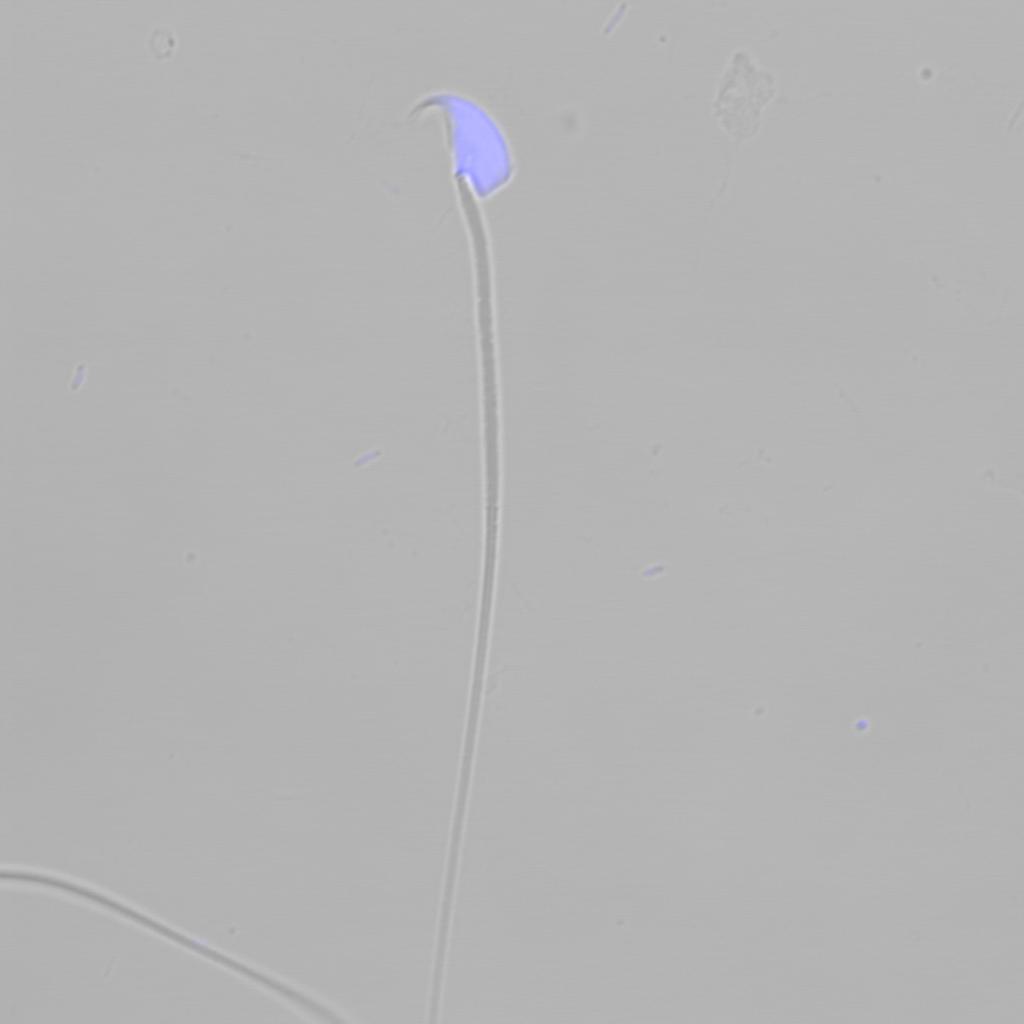

Supplement: Figure S4A Morphology of the cauda epididymal spermatozoa from Cfap52+/+ and Cfap52–/– mice. [file mmc2.zip › Figure 4A/Image 37 ok_c1+2+3.tif]

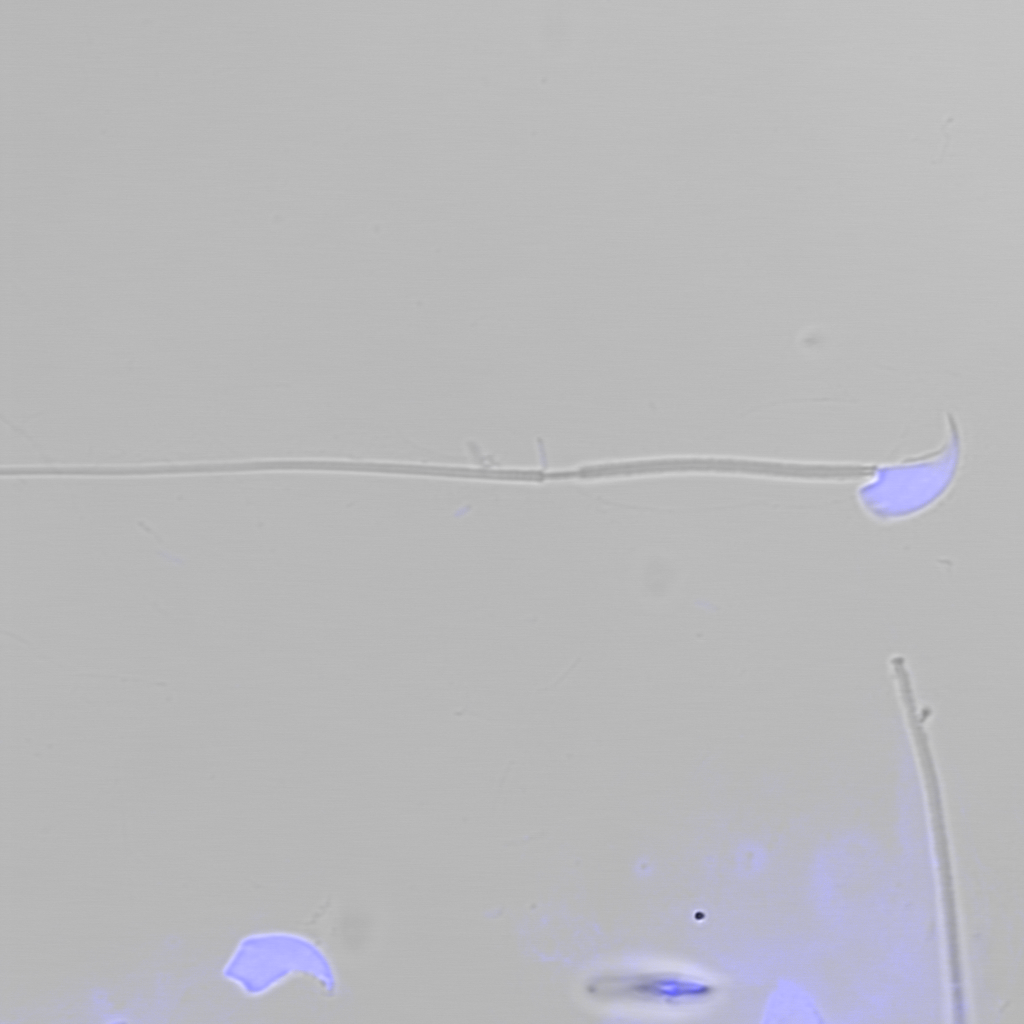

Supplement: Figure S4A Morphology of the cauda epididymal spermatozoa from Cfap52+/+ and Cfap52–/– mice. [file mmc2.zip › Figure 4A/ok_c1+2+3.tif]

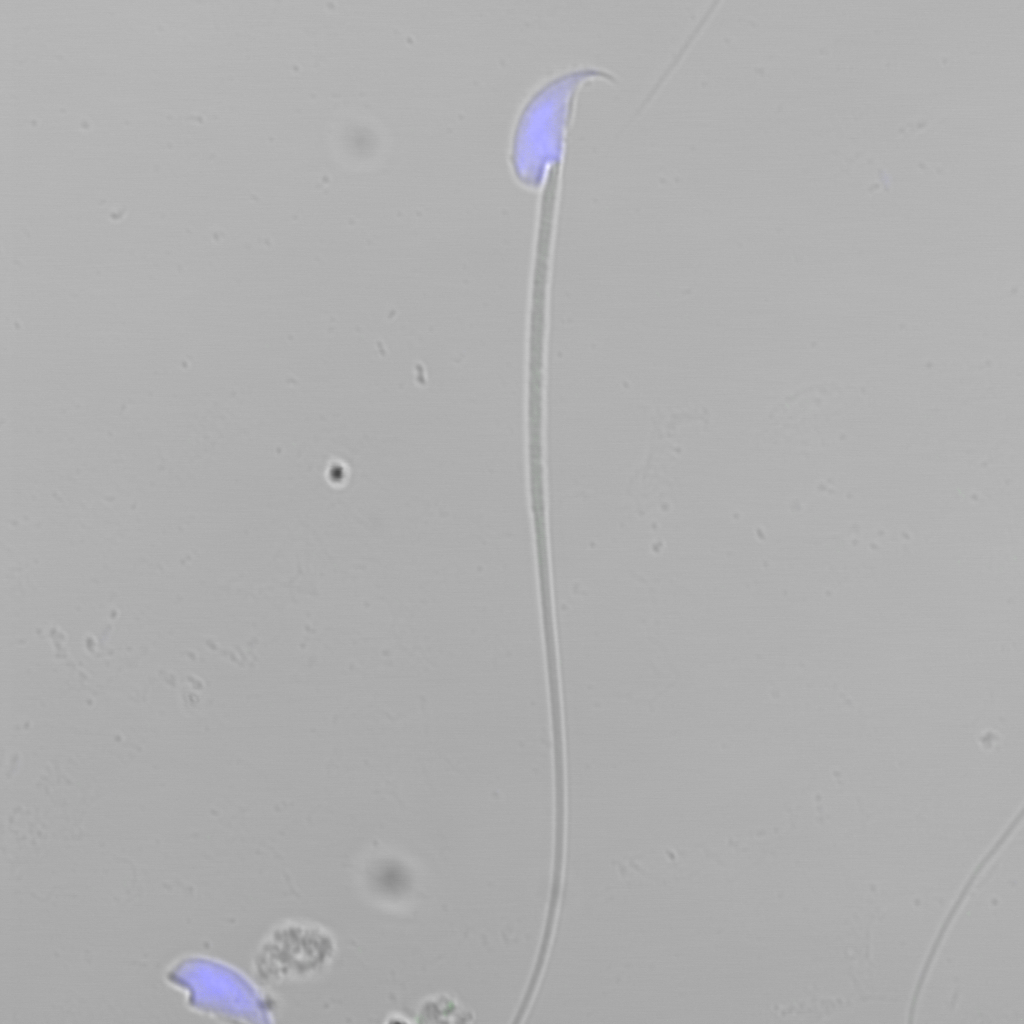

Supplement: Figure S4A Morphology of the cauda epididymal spermatozoa from Cfap52+/+ and Cfap52–/– mice. [file mmc2.zip › Figure 4A/wt 2_c1+2+3.tif]

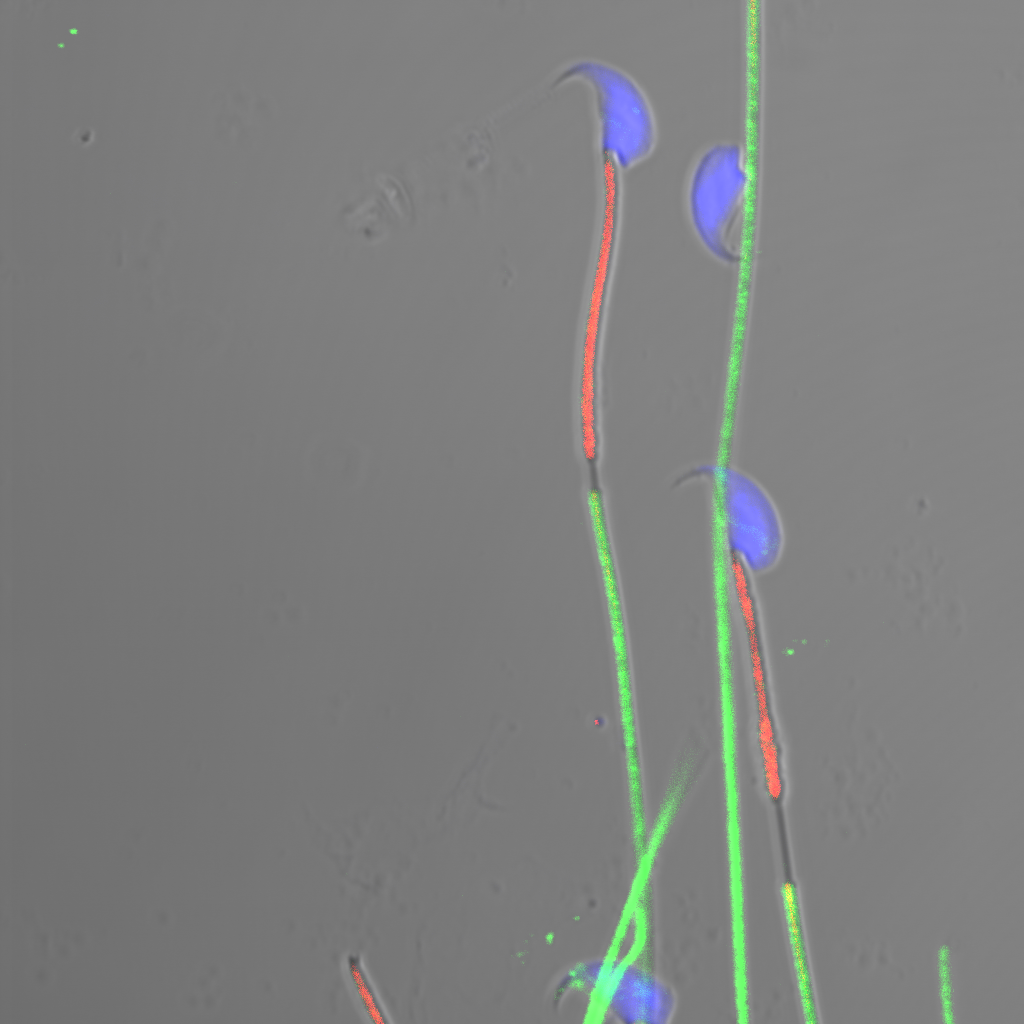

Supplement: Figure S4D Fluorescence staining of MitoTracker Deep Red and AKAP4 in Cfap52+/+ and Cfap52–/– spermatozoa; white arrowhead indicates disorganization of the midpiece-principal piece junction of the sperm tail. [file mmc3.zip › Figure 4D/Image 29_c1+2+3+4.tif]

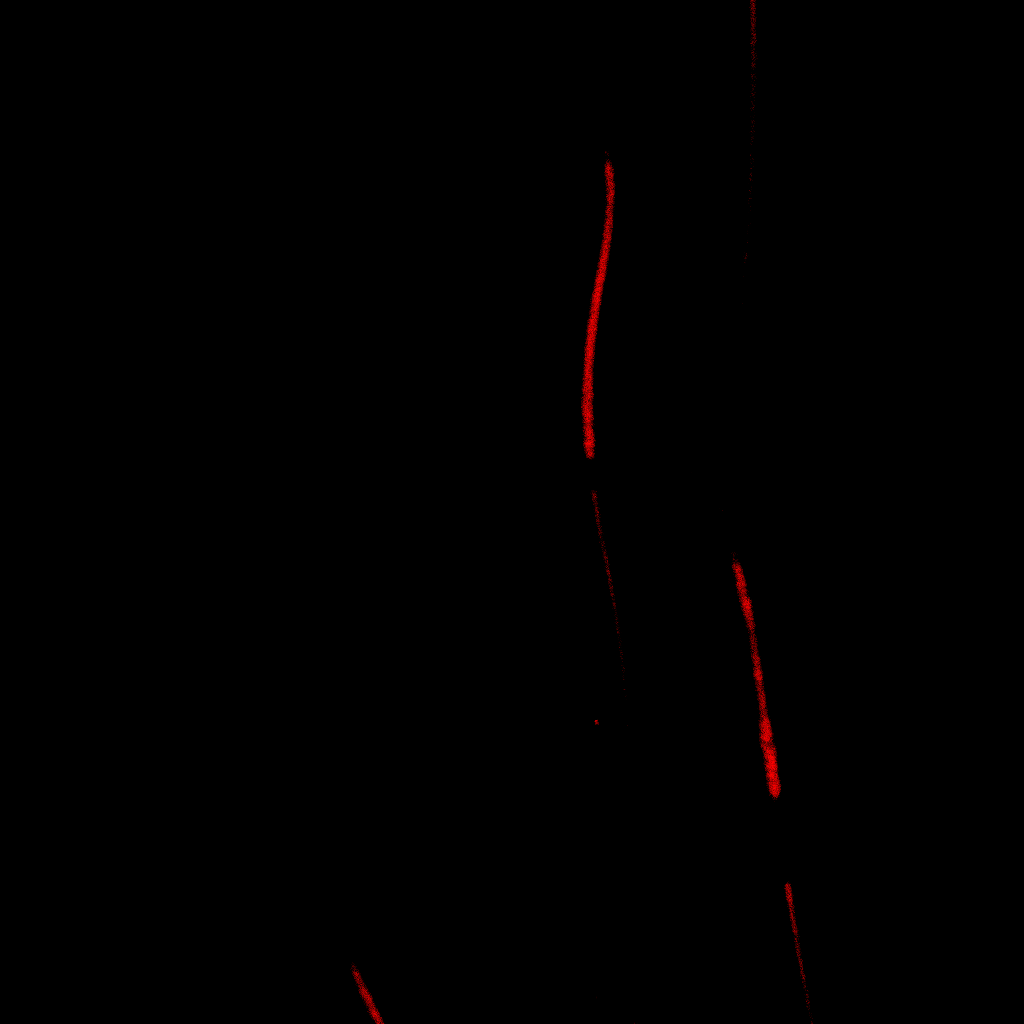

Supplement: Figure S4D Fluorescence staining of MitoTracker Deep Red and AKAP4 in Cfap52+/+ and Cfap52–/– spermatozoa; white arrowhead indicates disorganization of the midpiece-principal piece junction of the sperm tail. [file mmc3.zip › Figure 4D/Image 29_c1.tif]

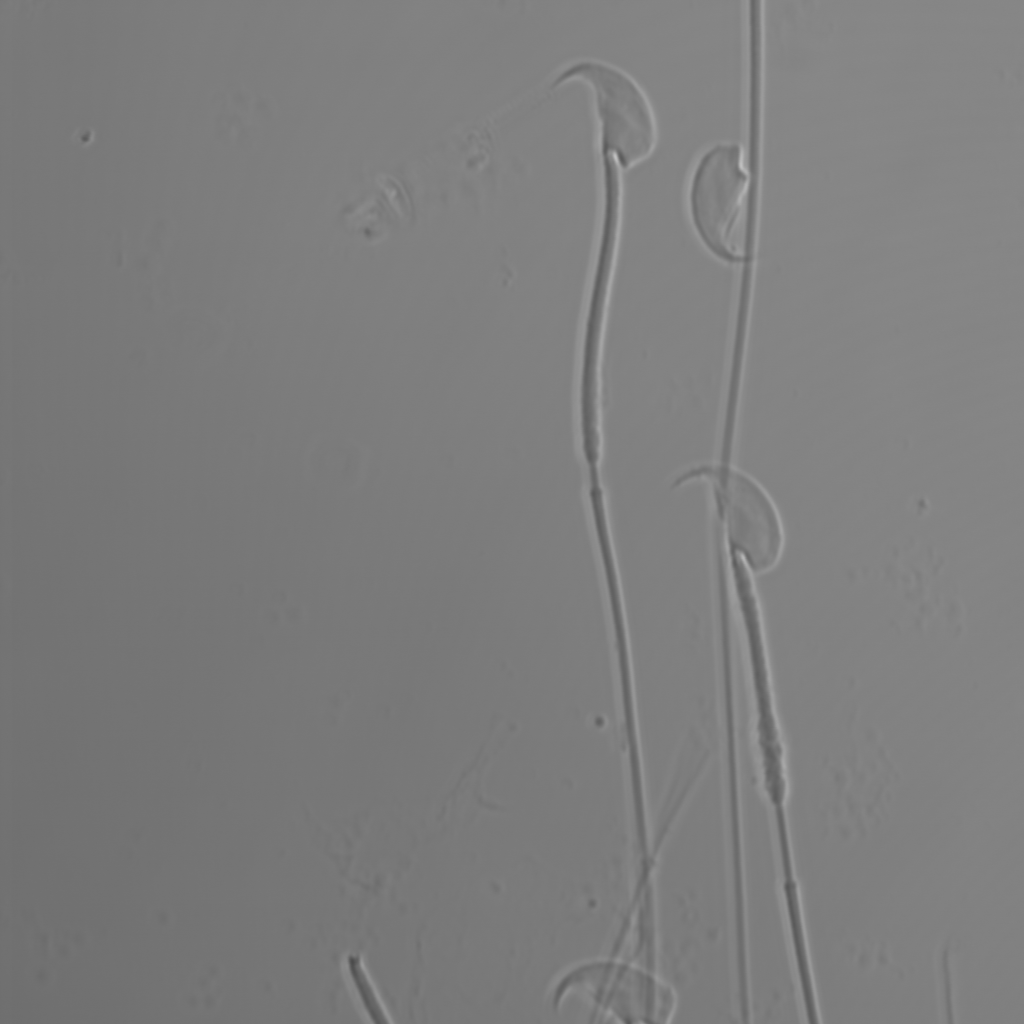

Supplement: Figure S4D Fluorescence staining of MitoTracker Deep Red and AKAP4 in Cfap52+/+ and Cfap52–/– spermatozoa; white arrowhead indicates disorganization of the midpiece-principal piece junction of the sperm tail. [file mmc3.zip › Figure 4D/Image 29_c2.tif]

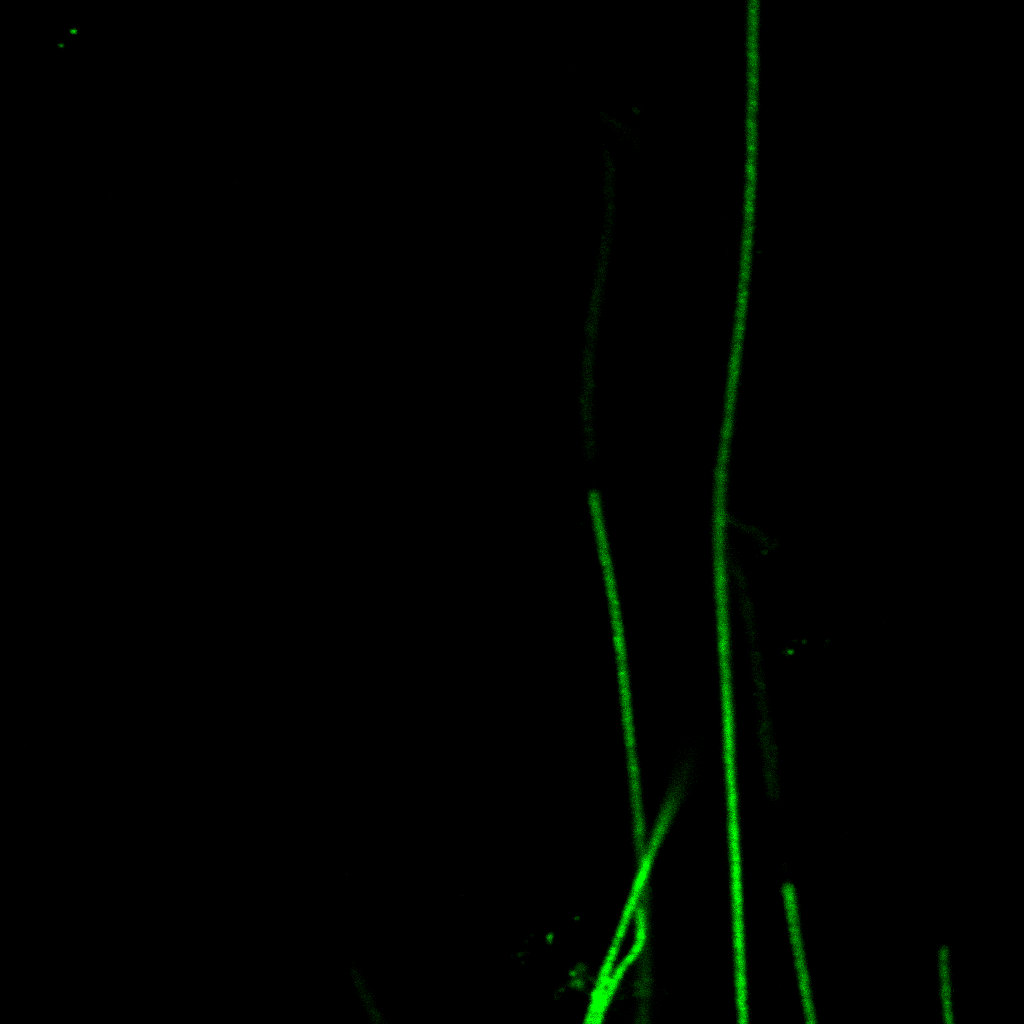

Supplement: Figure S4D Fluorescence staining of MitoTracker Deep Red and AKAP4 in Cfap52+/+ and Cfap52–/– spermatozoa; white arrowhead indicates disorganization of the midpiece-principal piece junction of the sperm tail. [file mmc3.zip › Figure 4D/Image 29_c3.tif]

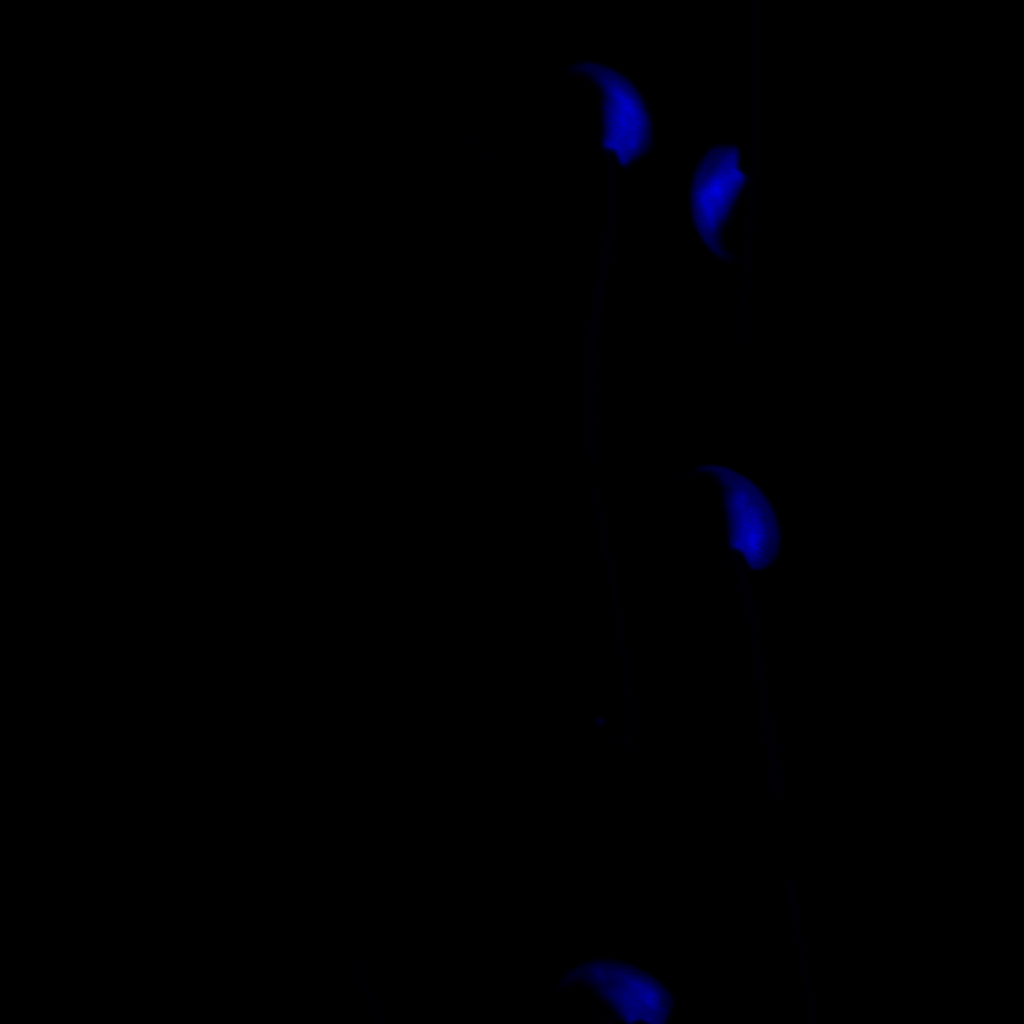

Supplement: Figure S4D Fluorescence staining of MitoTracker Deep Red and AKAP4 in Cfap52+/+ and Cfap52–/– spermatozoa; white arrowhead indicates disorganization of the midpiece-principal piece junction of the sperm tail. [file mmc3.zip › Figure 4D/Image 29_c4.tif]

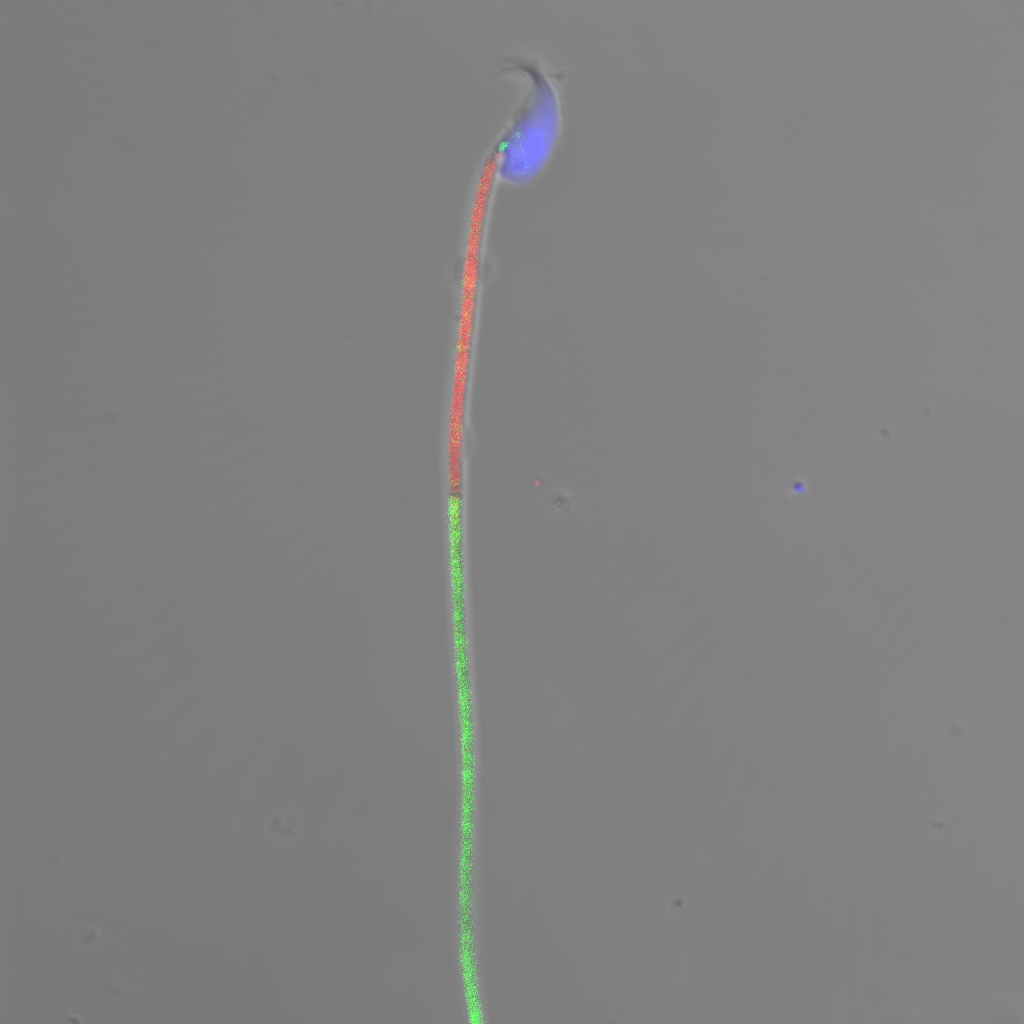

Supplement: Figure S4D Fluorescence staining of MitoTracker Deep Red and AKAP4 in Cfap52+/+ and Cfap52–/– spermatozoa; white arrowhead indicates disorganization of the midpiece-principal piece junction of the sperm tail. [file mmc3.zip › Figure 4D/wt_c1+2+3+4.tif]

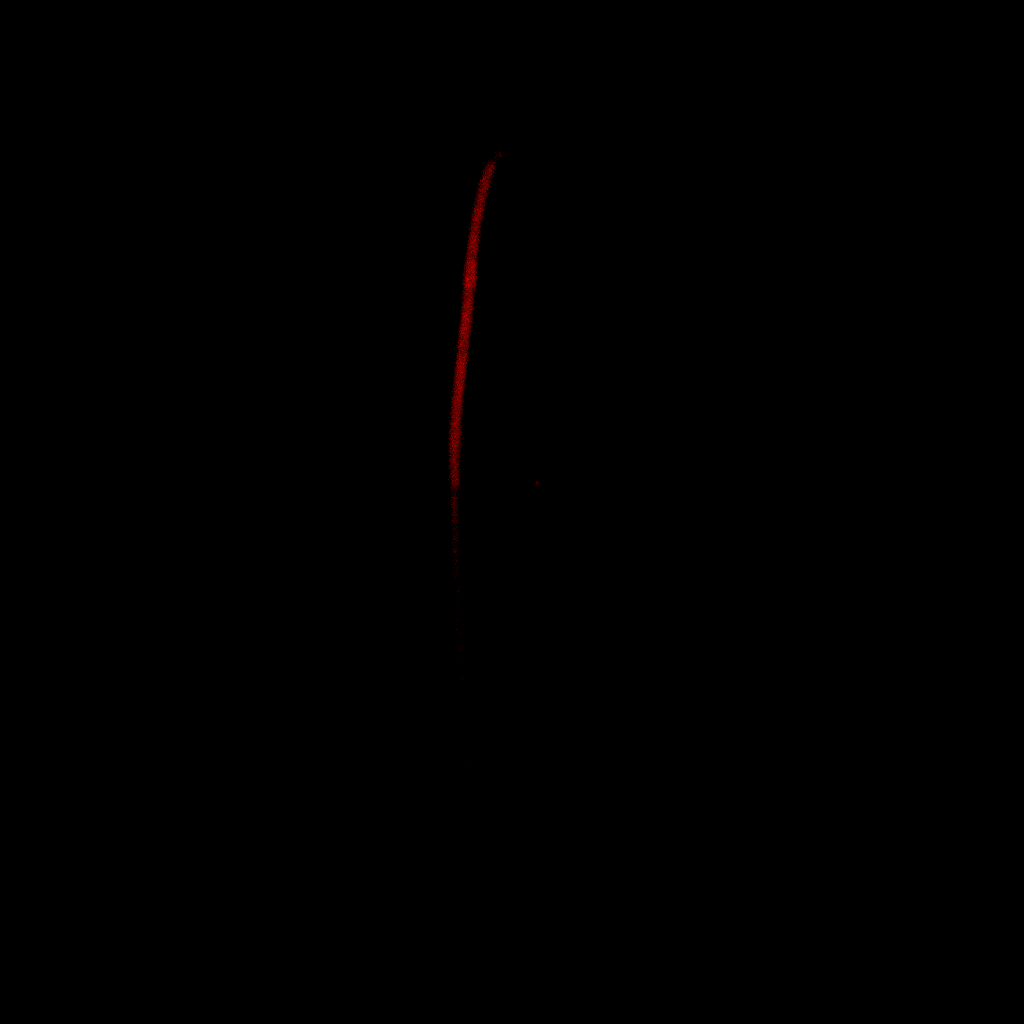

Supplement: Figure S4D Fluorescence staining of MitoTracker Deep Red and AKAP4 in Cfap52+/+ and Cfap52–/– spermatozoa; white arrowhead indicates disorganization of the midpiece-principal piece junction of the sperm tail. [file mmc3.zip › Figure 4D/wt_c1.tif]

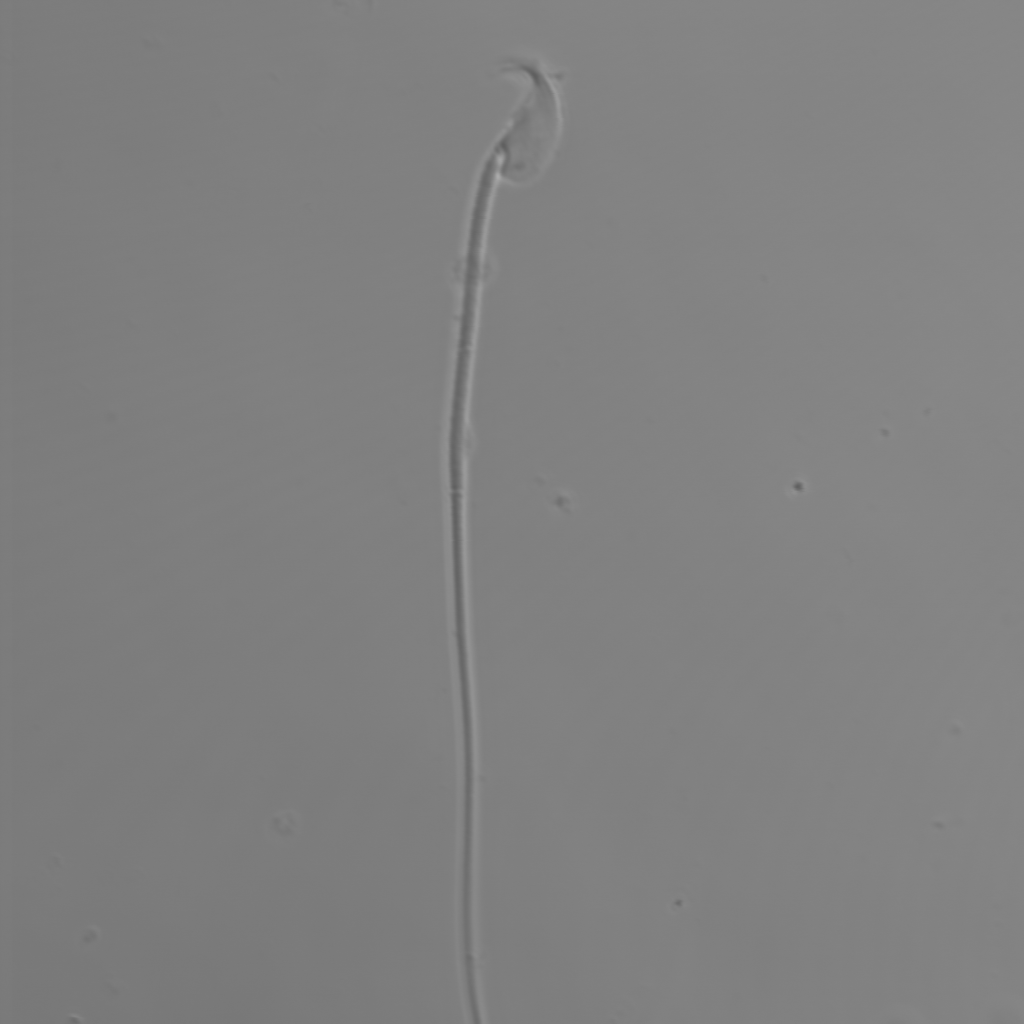

Supplement: Figure S4D Fluorescence staining of MitoTracker Deep Red and AKAP4 in Cfap52+/+ and Cfap52–/– spermatozoa; white arrowhead indicates disorganization of the midpiece-principal piece junction of the sperm tail. [file mmc3.zip › Figure 4D/wt_c2.tif]

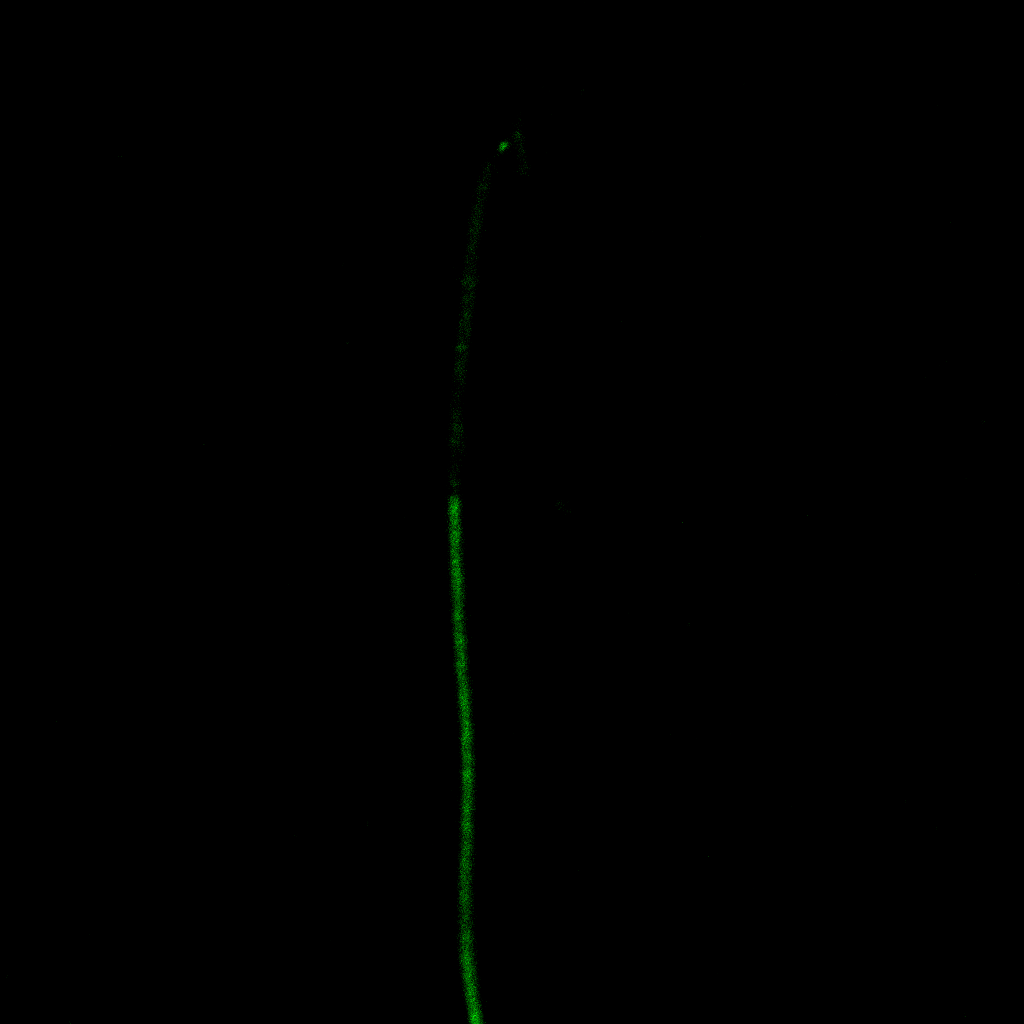

Supplement: Figure S4D Fluorescence staining of MitoTracker Deep Red and AKAP4 in Cfap52+/+ and Cfap52–/– spermatozoa; white arrowhead indicates disorganization of the midpiece-principal piece junction of the sperm tail. [file mmc3.zip › Figure 4D/wt_c3.tif]

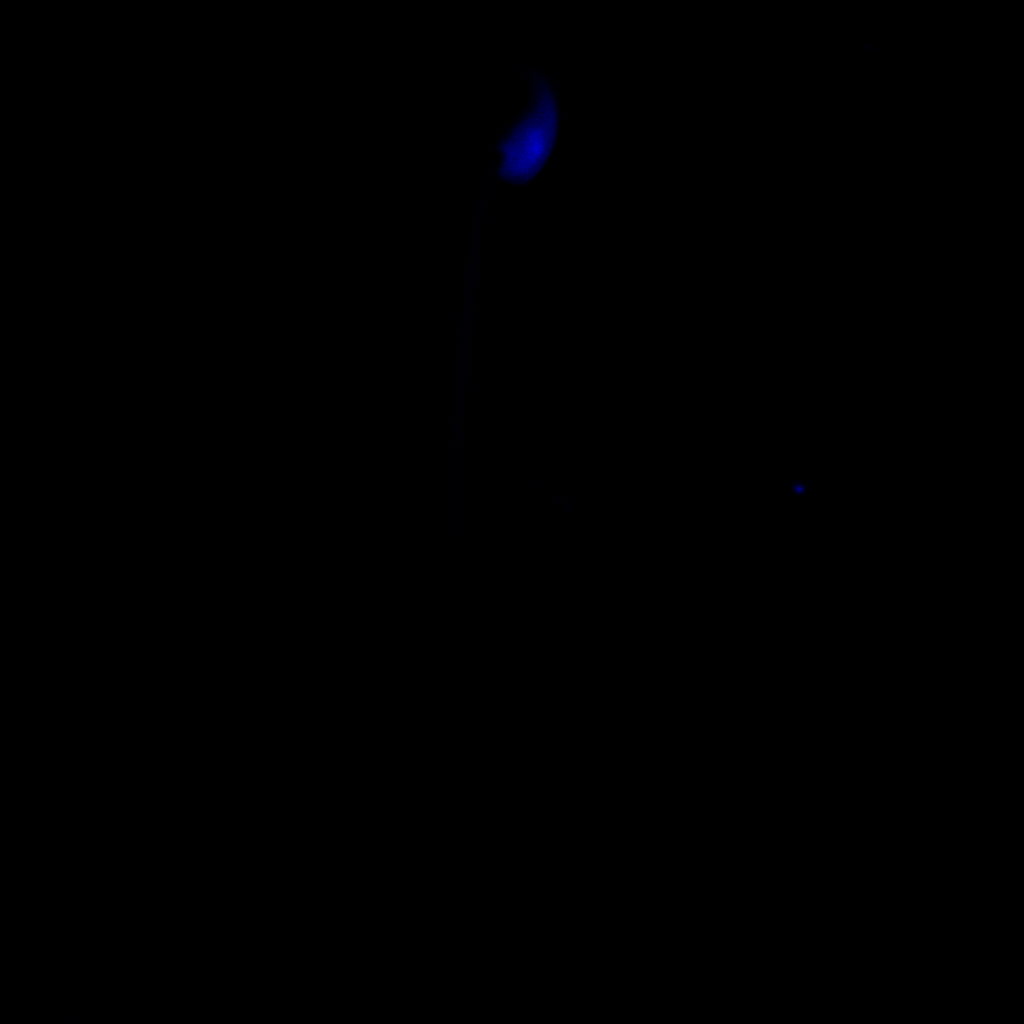

Supplement: Figure S4D Fluorescence staining of MitoTracker Deep Red and AKAP4 in Cfap52+/+ and Cfap52–/– spermatozoa; white arrowhead indicates disorganization of the midpiece-principal piece junction of the sperm tail. [file mmc3.zip › Figure 4D/wt_c4.tif]

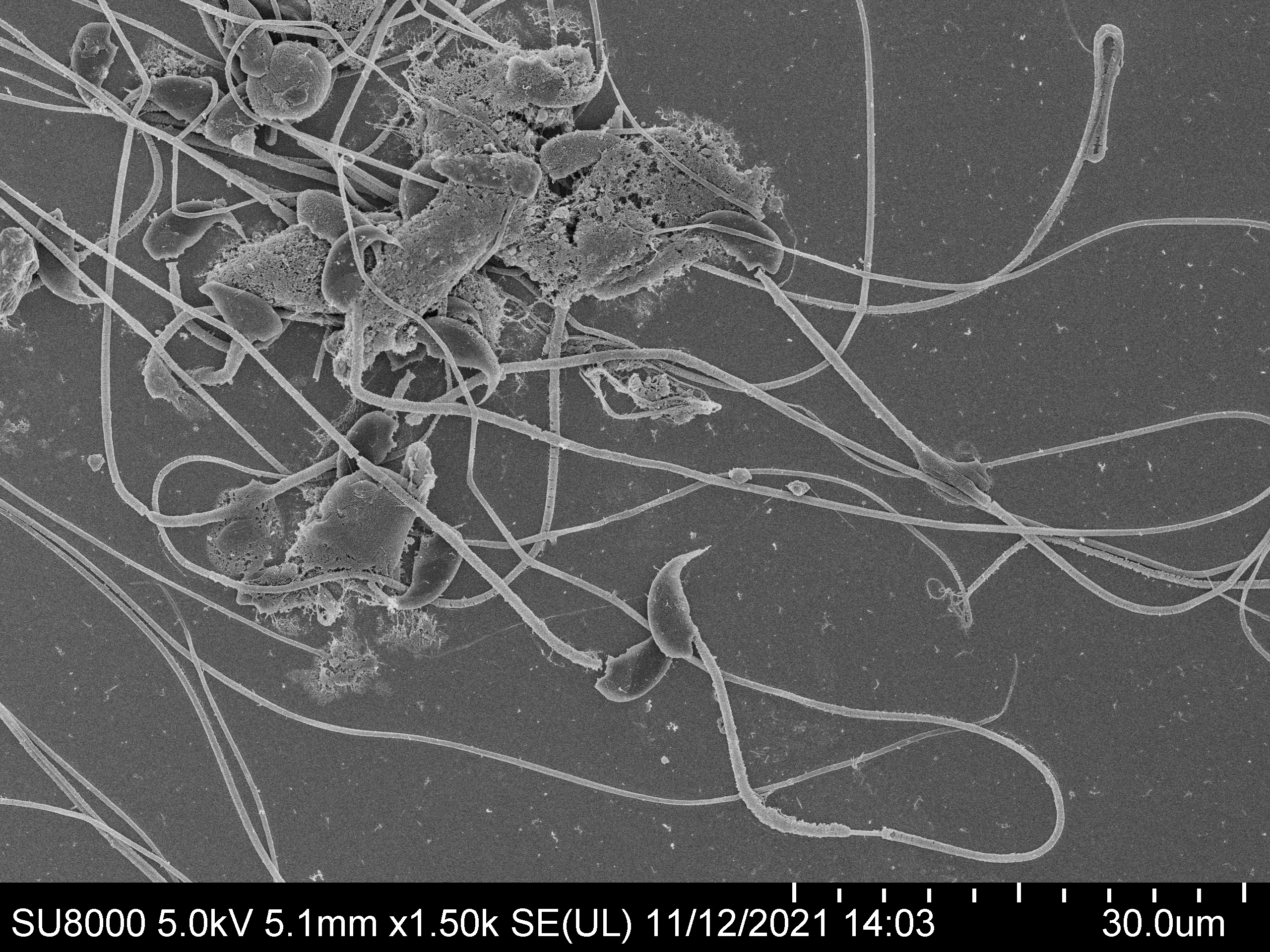

Supplement: Figure S5A Scanning electron microscope (SEM) analysis of spermatozoa from the cauda epididymidis of Cfap52+/+ and Cfap52-/- mice. Magnified images (white boxes) are shown in the lower panels. [file mmc4.zip › Figure 5A/KO_m002.tif]

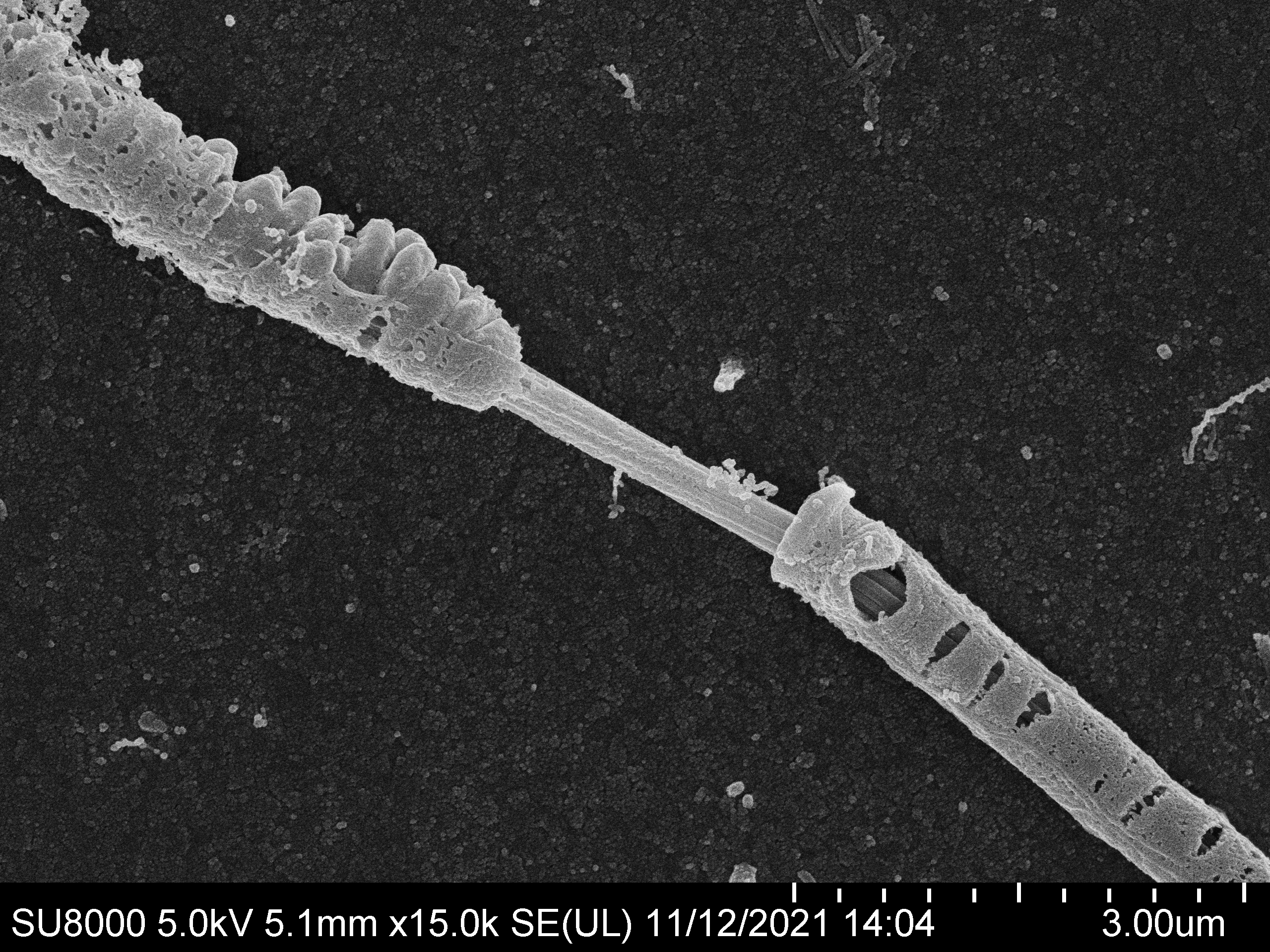

Supplement: Figure S5A Scanning electron microscope (SEM) analysis of spermatozoa from the cauda epididymidis of Cfap52+/+ and Cfap52-/- mice. Magnified images (white boxes) are shown in the lower panels. [file mmc4.zip › Figure 5A/KO_m003.tif]

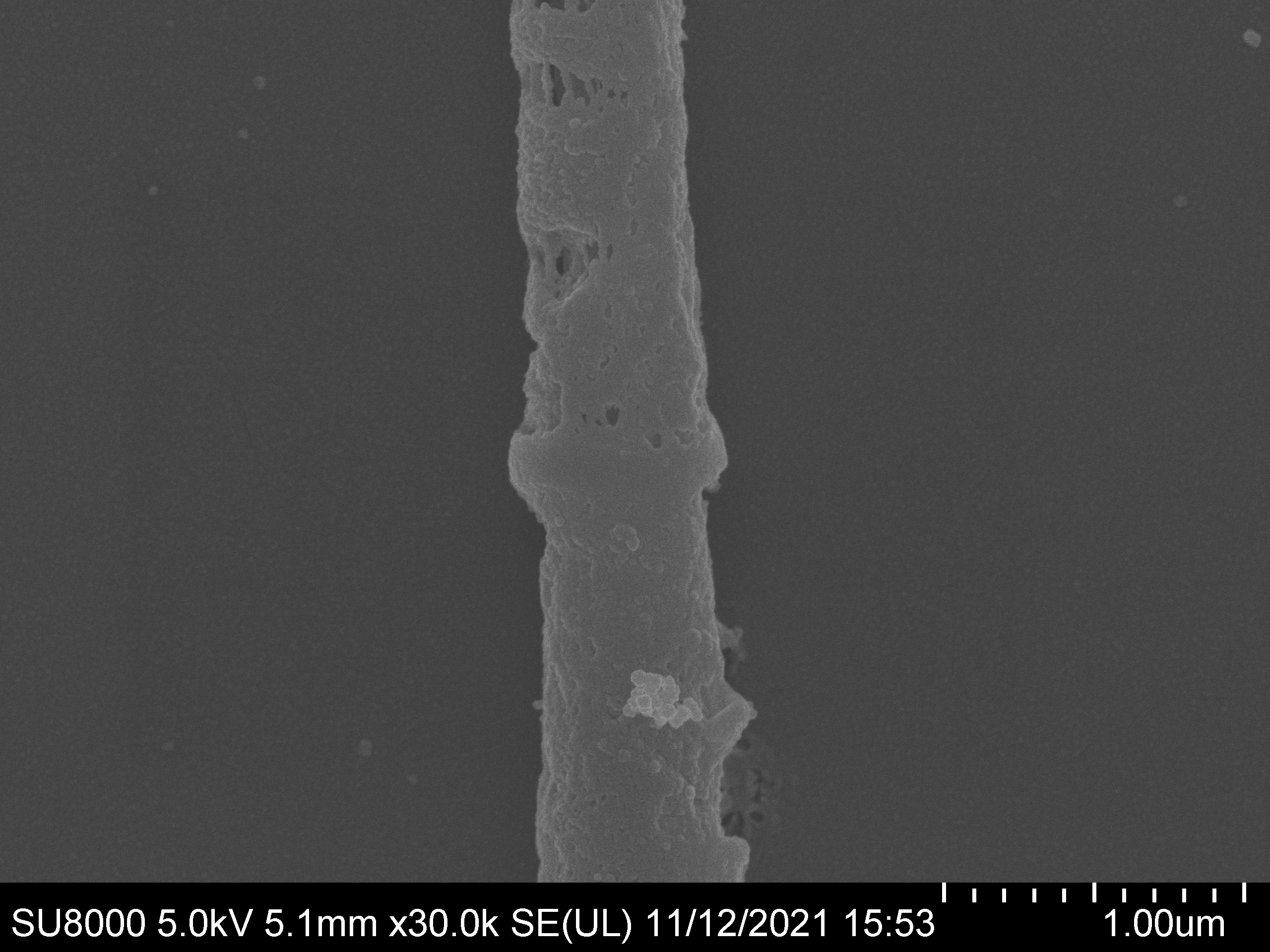

Supplement: Figure S5A Scanning electron microscope (SEM) analysis of spermatozoa from the cauda epididymidis of Cfap52+/+ and Cfap52-/- mice. Magnified images (white boxes) are shown in the lower panels. [file mmc4.zip › Figure 5A/m013.tif]

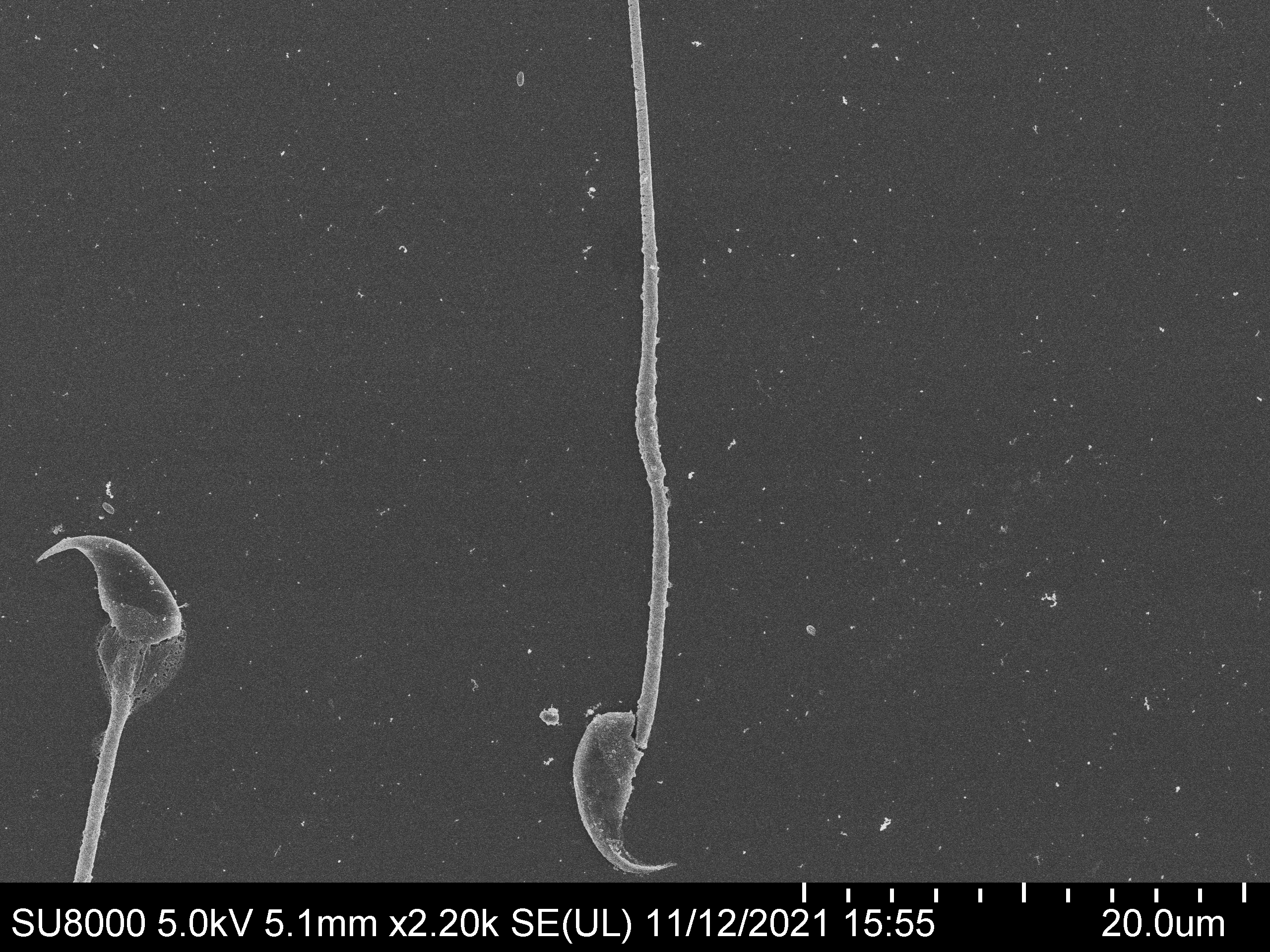

Supplement: Figure S5A Scanning electron microscope (SEM) analysis of spermatozoa from the cauda epididymidis of Cfap52+/+ and Cfap52-/- mice. Magnified images (white boxes) are shown in the lower panels. [file mmc4.zip › Figure 5A/m014.tif]

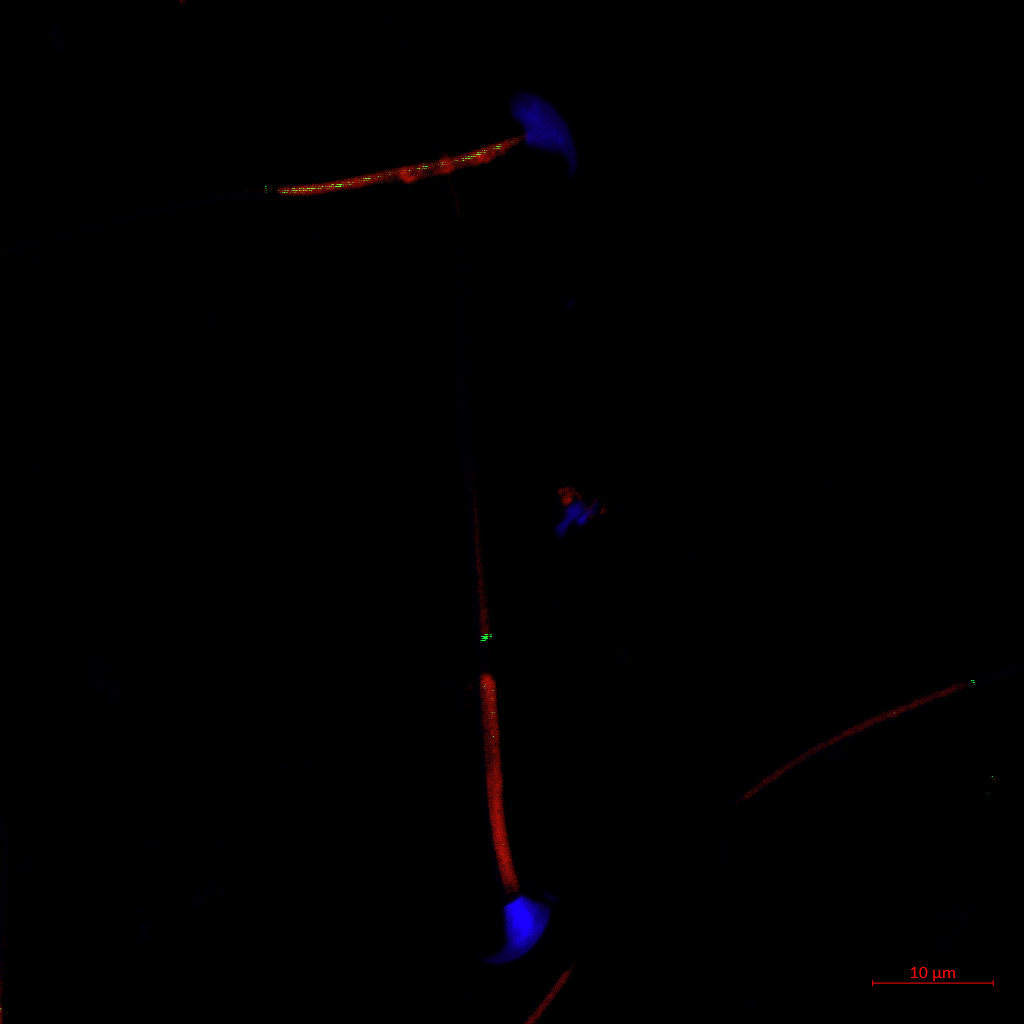

Supplement: Figure S5C Fluorescence staining of MitoTracker Deep Red and SEPT4 in Cfap52+/+ and Cfap52–/– spermatozoa; white arrowhead indicates the annulus region. [file mmc6.zip › Figure 5C/52 KO MITO SEPT4 Snap-2228/Snap-2228_c1+2+3+4.tif]

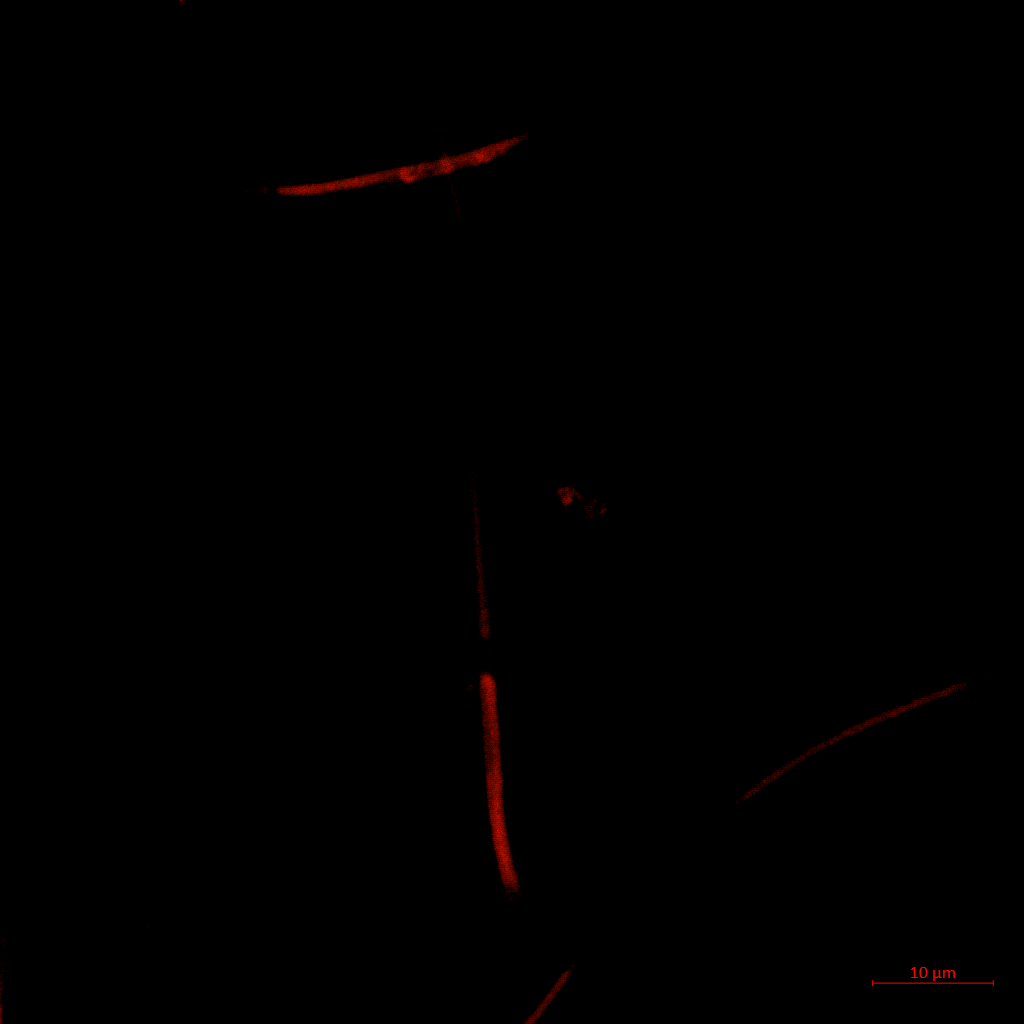

Supplement: Figure S5C Fluorescence staining of MitoTracker Deep Red and SEPT4 in Cfap52+/+ and Cfap52–/– spermatozoa; white arrowhead indicates the annulus region. [file mmc6.zip › Figure 5C/52 KO MITO SEPT4 Snap-2228/Snap-2228_c1.tif]

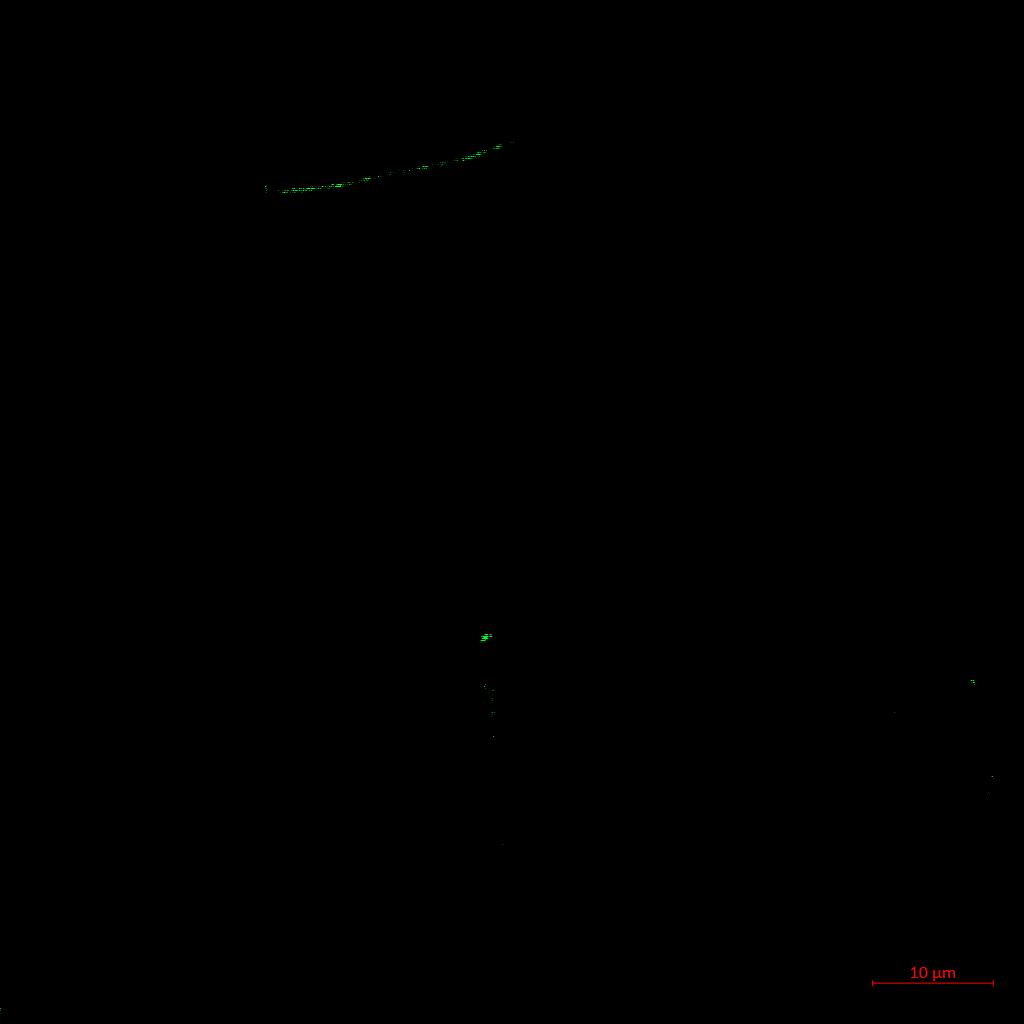

Supplement: Figure S5C Fluorescence staining of MitoTracker Deep Red and SEPT4 in Cfap52+/+ and Cfap52–/– spermatozoa; white arrowhead indicates the annulus region. [file mmc6.zip › Figure 5C/52 KO MITO SEPT4 Snap-2228/Snap-2228_c2.tif]

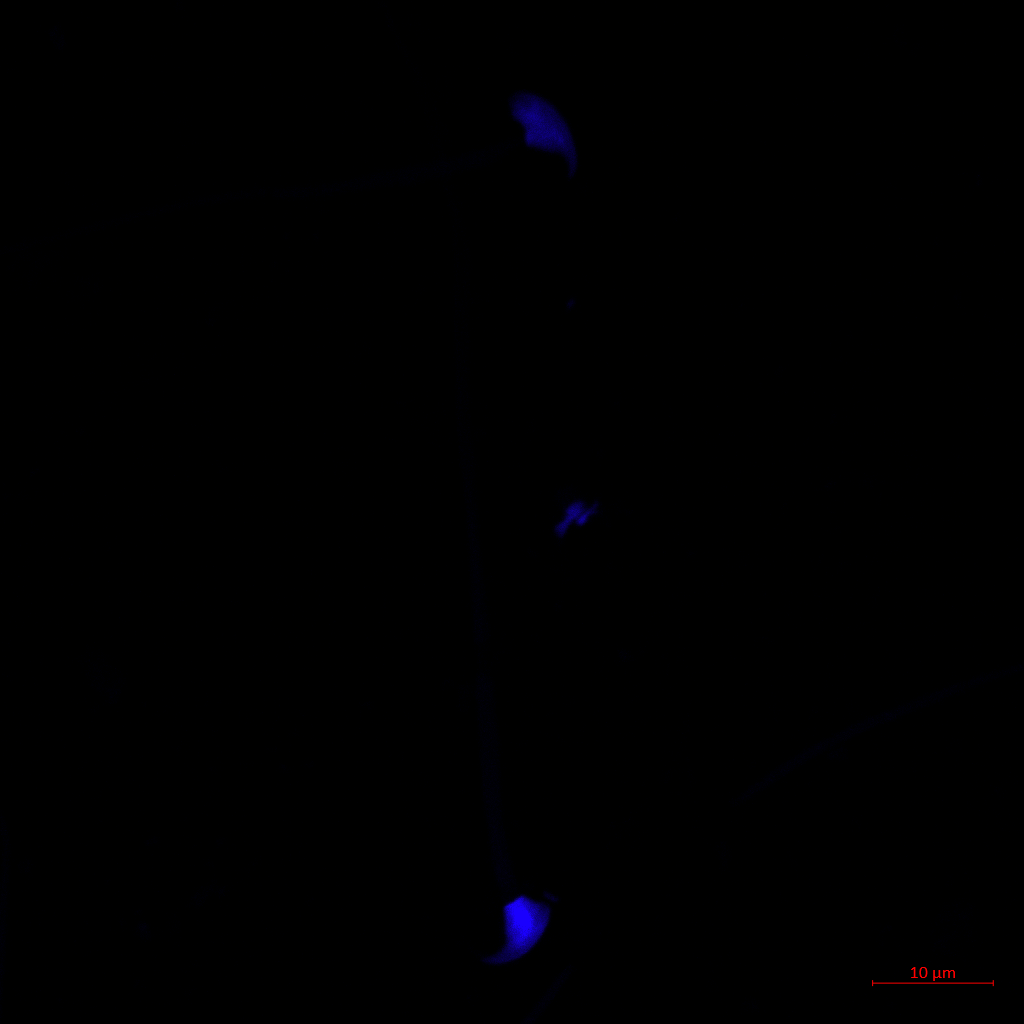

Supplement: Figure S5C Fluorescence staining of MitoTracker Deep Red and SEPT4 in Cfap52+/+ and Cfap52–/– spermatozoa; white arrowhead indicates the annulus region. [file mmc6.zip › Figure 5C/52 KO MITO SEPT4 Snap-2228/Snap-2228_c4.tif]

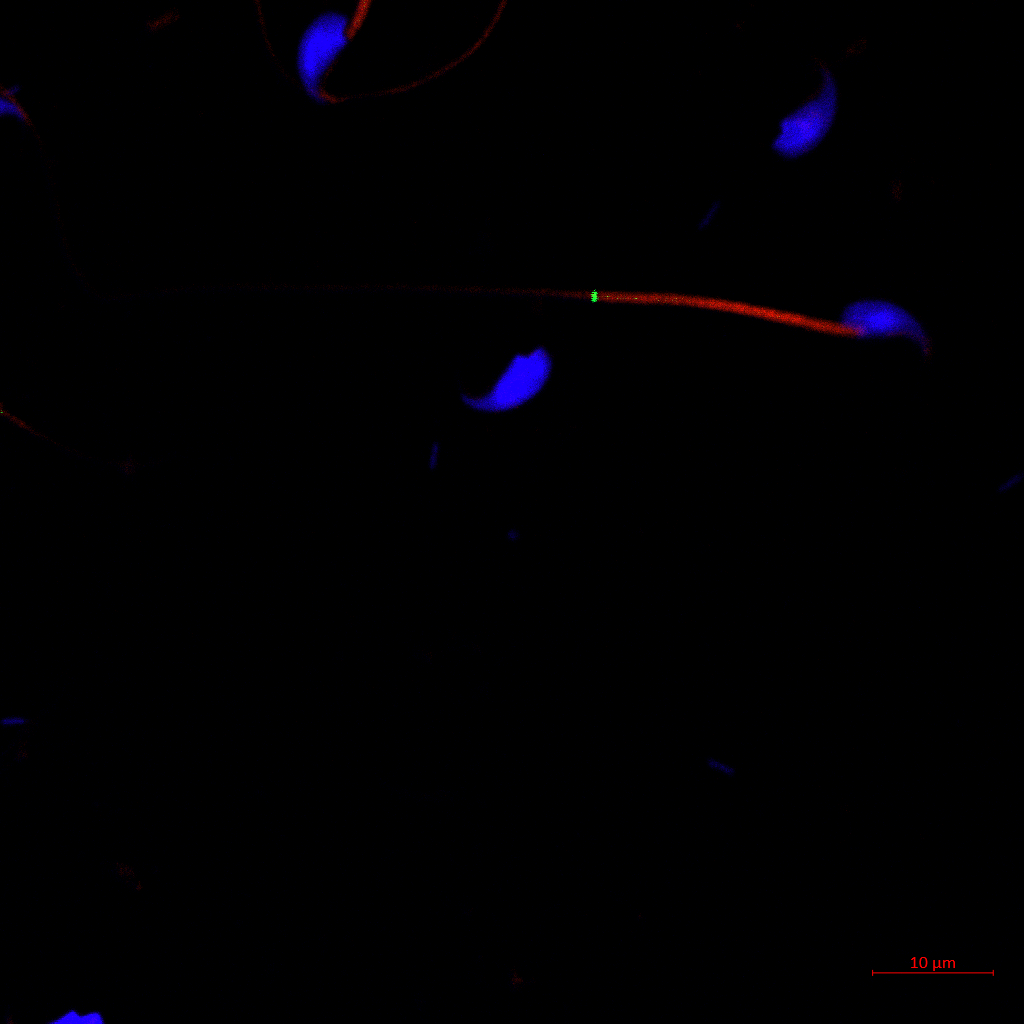

Supplement: Figure S5C Fluorescence staining of MitoTracker Deep Red and SEPT4 in Cfap52+/+ and Cfap52–/– spermatozoa; white arrowhead indicates the annulus region. [file mmc6.zip › Figure 5C/52 WT MITO SEPT4/Snap-2236_c1+2+3+4.tif]

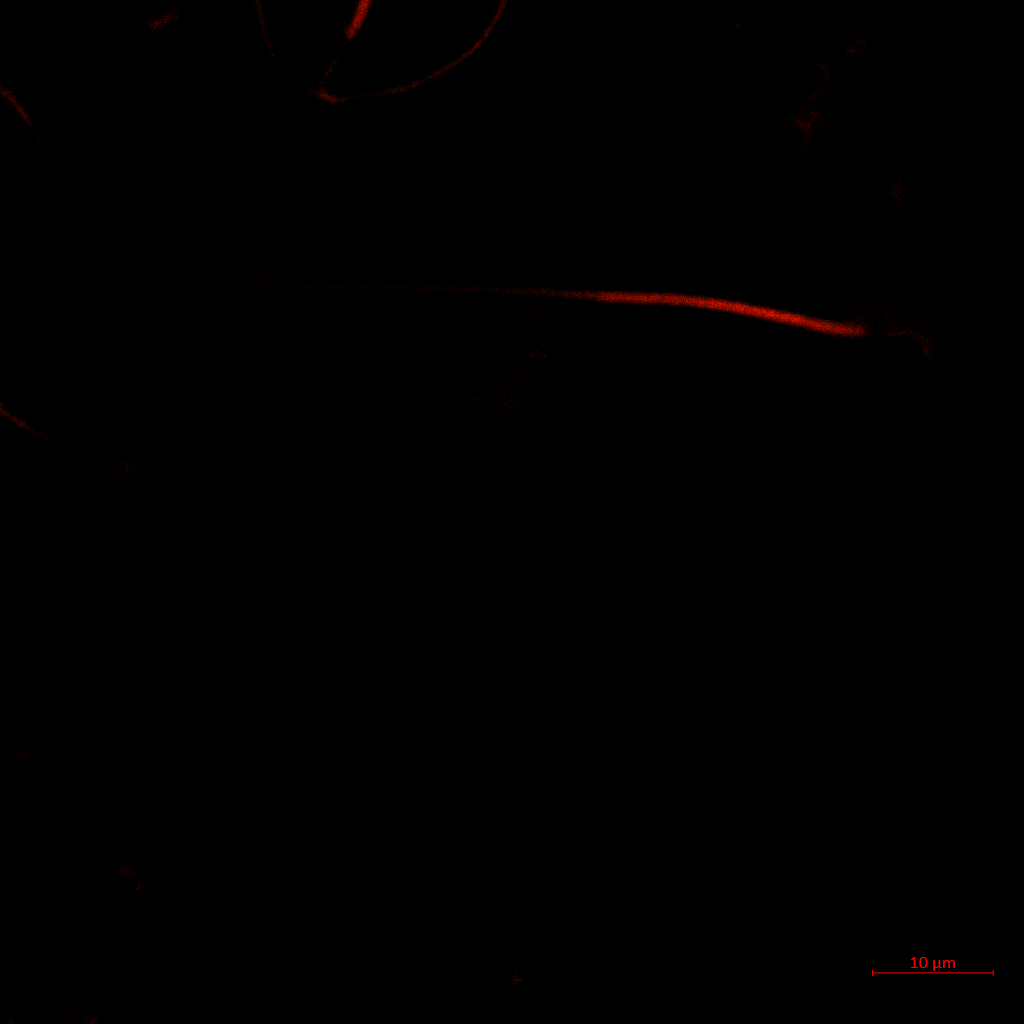

Supplement: Figure S5C Fluorescence staining of MitoTracker Deep Red and SEPT4 in Cfap52+/+ and Cfap52–/– spermatozoa; white arrowhead indicates the annulus region. [file mmc6.zip › Figure 5C/52 WT MITO SEPT4/Snap-2236_c1.tif]

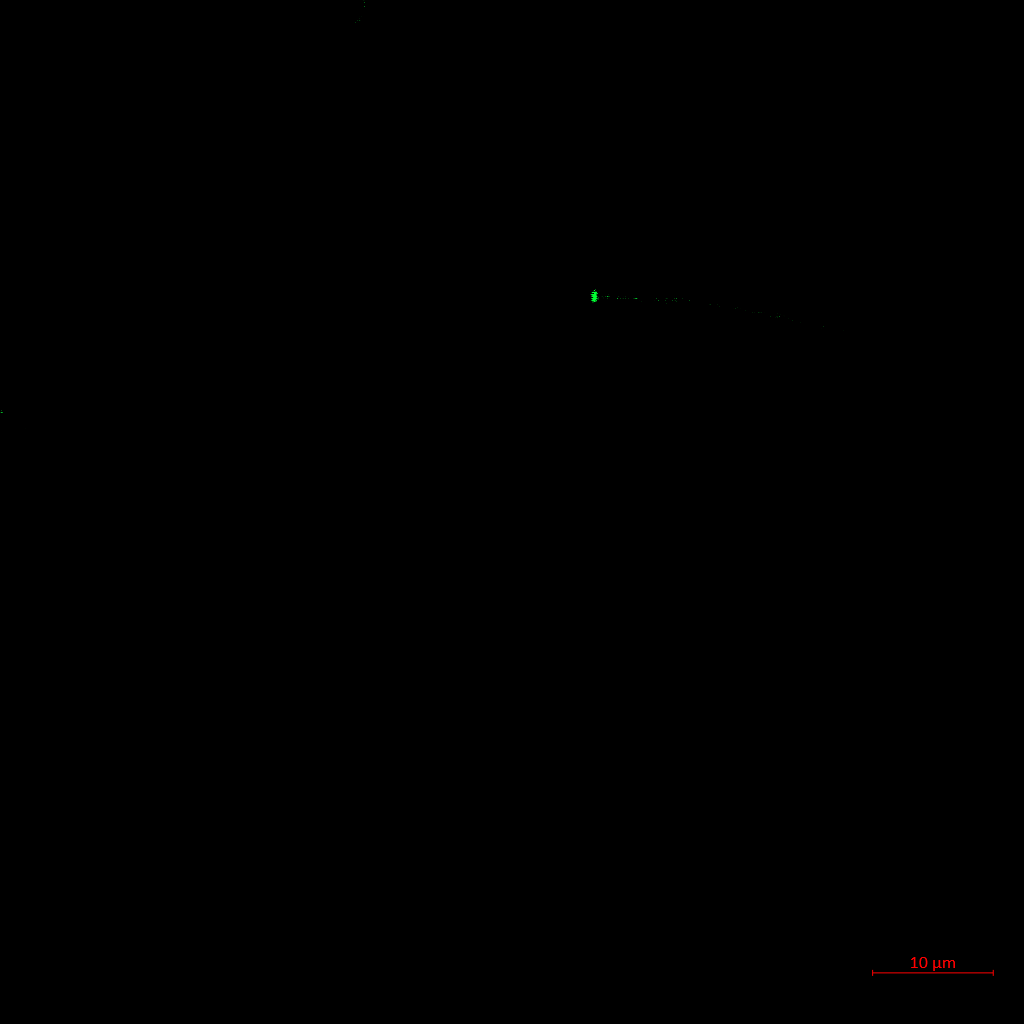

Supplement: Figure S5C Fluorescence staining of MitoTracker Deep Red and SEPT4 in Cfap52+/+ and Cfap52–/– spermatozoa; white arrowhead indicates the annulus region. [file mmc6.zip › Figure 5C/52 WT MITO SEPT4/Snap-2236_c2.tif]

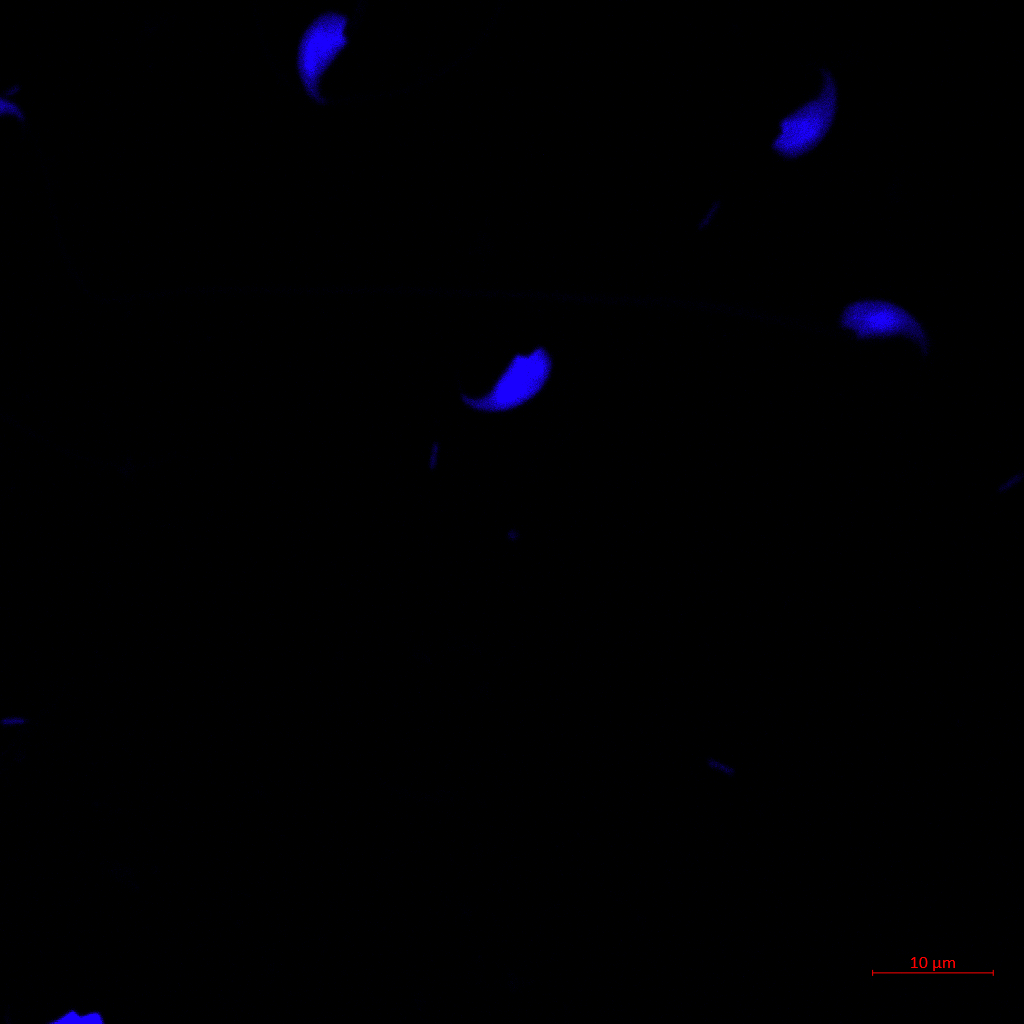

Supplement: Figure S5C Fluorescence staining of MitoTracker Deep Red and SEPT4 in Cfap52+/+ and Cfap52–/– spermatozoa; white arrowhead indicates the annulus region. [file mmc6.zip › Figure 5C/52 WT MITO SEPT4/Snap-2236_c4.tif]

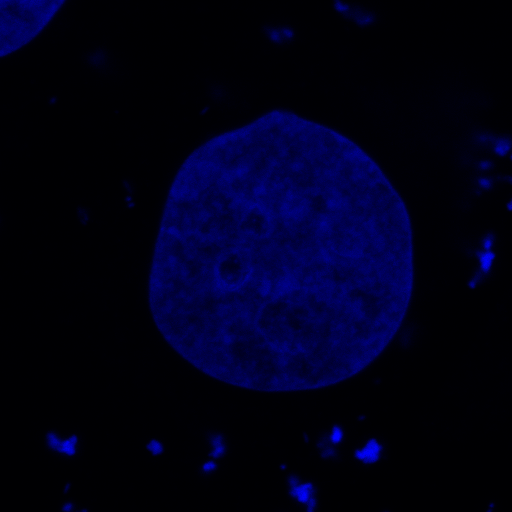

Supplement: Figure S6C CFAP52 colocalized with CFAP45 in HeLa cells. pCS2-MYC-CFAP45 and pEGFP-GFP-CFAP52 plasmids were cotransfected into HeLa cells. Twenty four hours after transfection, the cells were fixed and stained with anti-MYC and anti-GFP antibodies, and the nuclei were stained with DAPI (blue). [file mmc7.zip › Figure 6C/gfp 52 myc 45.lif_Series014_Crop001_Processed001_ch00.tif]

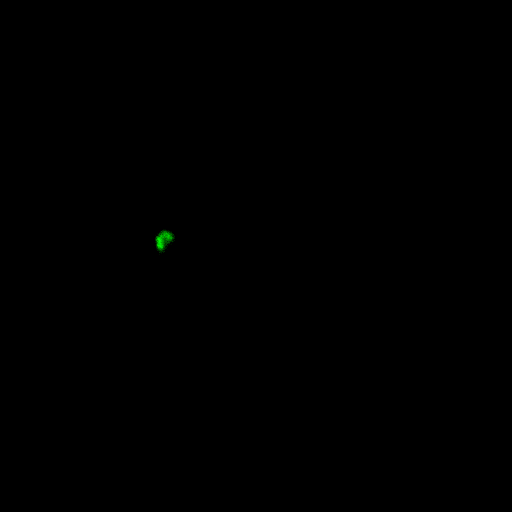

Supplement: Figure S6C CFAP52 colocalized with CFAP45 in HeLa cells. pCS2-MYC-CFAP45 and pEGFP-GFP-CFAP52 plasmids were cotransfected into HeLa cells. Twenty four hours after transfection, the cells were fixed and stained with anti-MYC and anti-GFP antibodies, and the nuclei were stained with DAPI (blue). [file mmc7.zip › Figure 6C/gfp 52 myc 45.lif_Series014_Crop002_z1_ch01.tif]

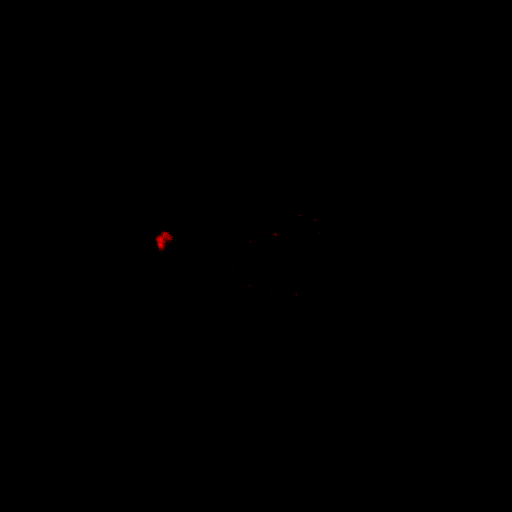

Supplement: Figure S6C CFAP52 colocalized with CFAP45 in HeLa cells. pCS2-MYC-CFAP45 and pEGFP-GFP-CFAP52 plasmids were cotransfected into HeLa cells. Twenty four hours after transfection, the cells were fixed and stained with anti-MYC and anti-GFP antibodies, and the nuclei were stained with DAPI (blue). [file mmc7.zip › Figure 6C/gfp 52 myc 45.lif_Series014_Crop002_z1_ch02.tif]

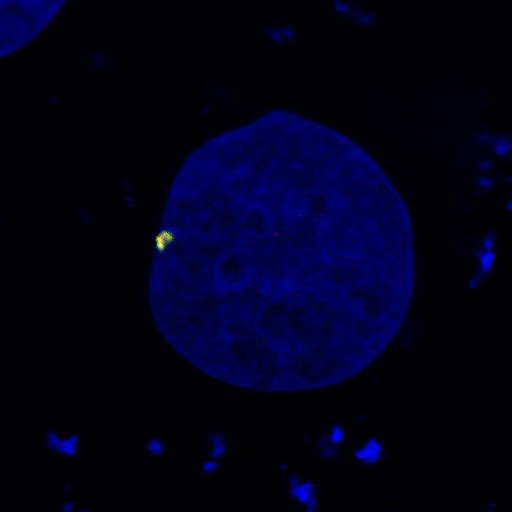

Supplement: Figure S6C CFAP52 colocalized with CFAP45 in HeLa cells. pCS2-MYC-CFAP45 and pEGFP-GFP-CFAP52 plasmids were cotransfected into HeLa cells. Twenty four hours after transfection, the cells were fixed and stained with anti-MYC and anti-GFP antibodies, and the nuclei were stained with DAPI (blue). [file mmc7.zip › Figure 6C/merge gfp 52 myc 45.lif_Series014_Crop001_Processed001_ch00.tif]

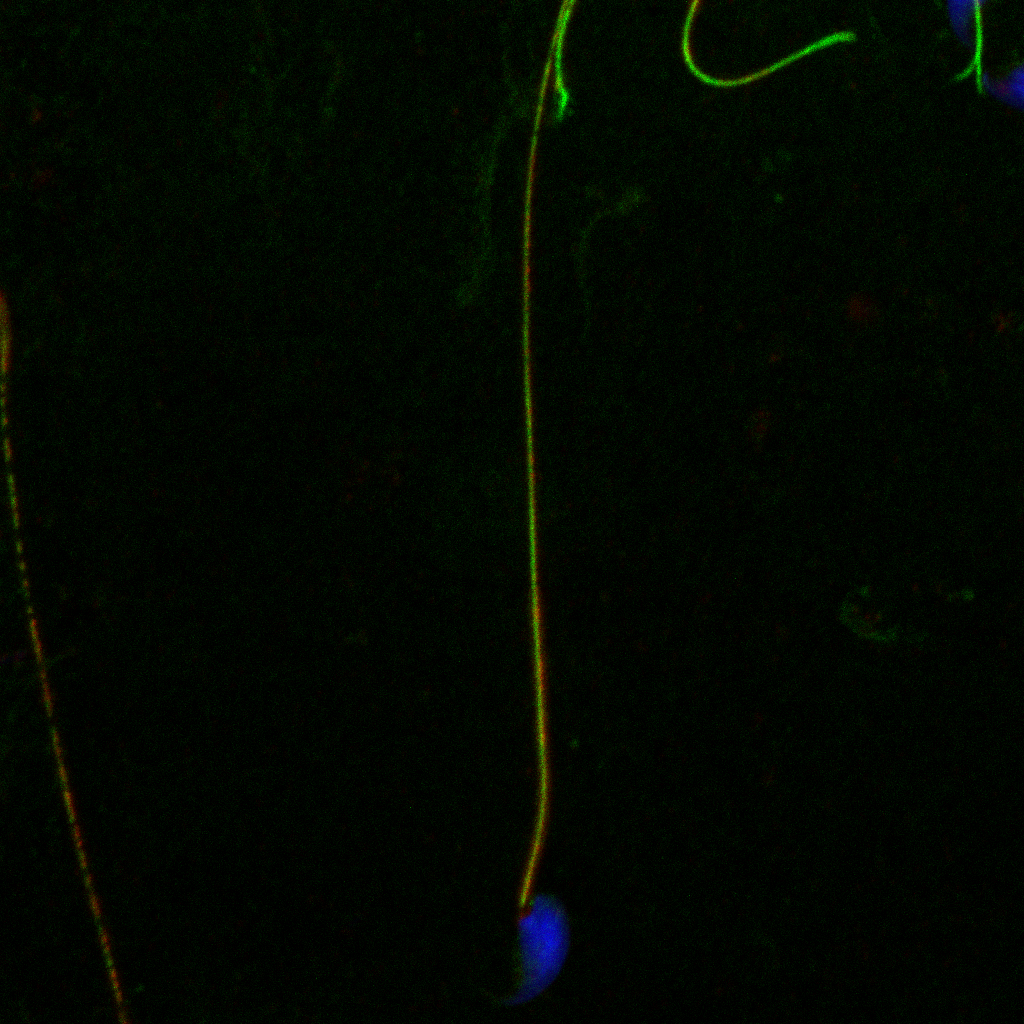

Supplement: Figure S6F Immunofluorescence of AcTub (green) and CFAP45 (red) in spermatozoa released from the caudal epididymis of Cfap52+/+ and Cfap52–/– mice. Nuclei were stained with DAPI (blue). [file mmc8.zip › Figure 6F/Image 56/Image 56_c1+2+3+4.tif]

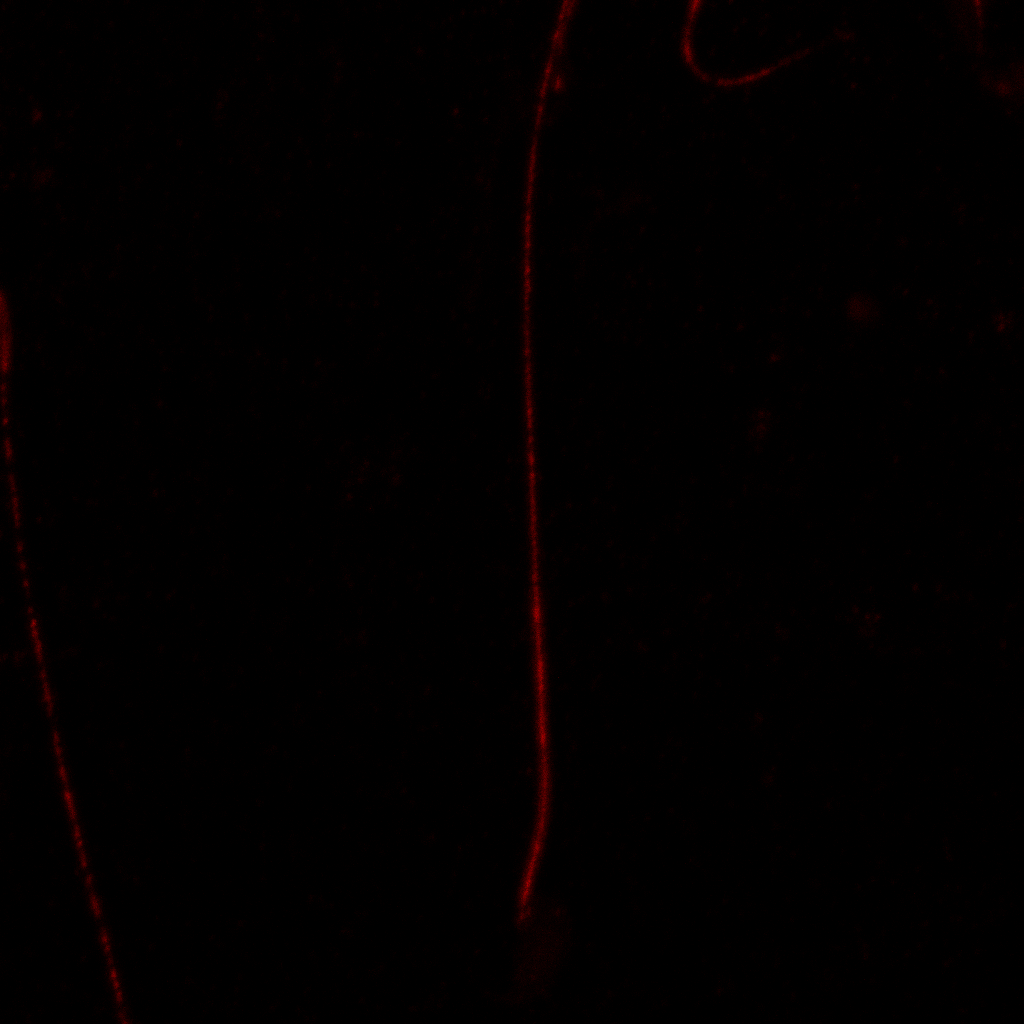

Supplement: Figure S6F Immunofluorescence of AcTub (green) and CFAP45 (red) in spermatozoa released from the caudal epididymis of Cfap52+/+ and Cfap52–/– mice. Nuclei were stained with DAPI (blue). [file mmc8.zip › Figure 6F/Image 56/Image 56_c1.tif]

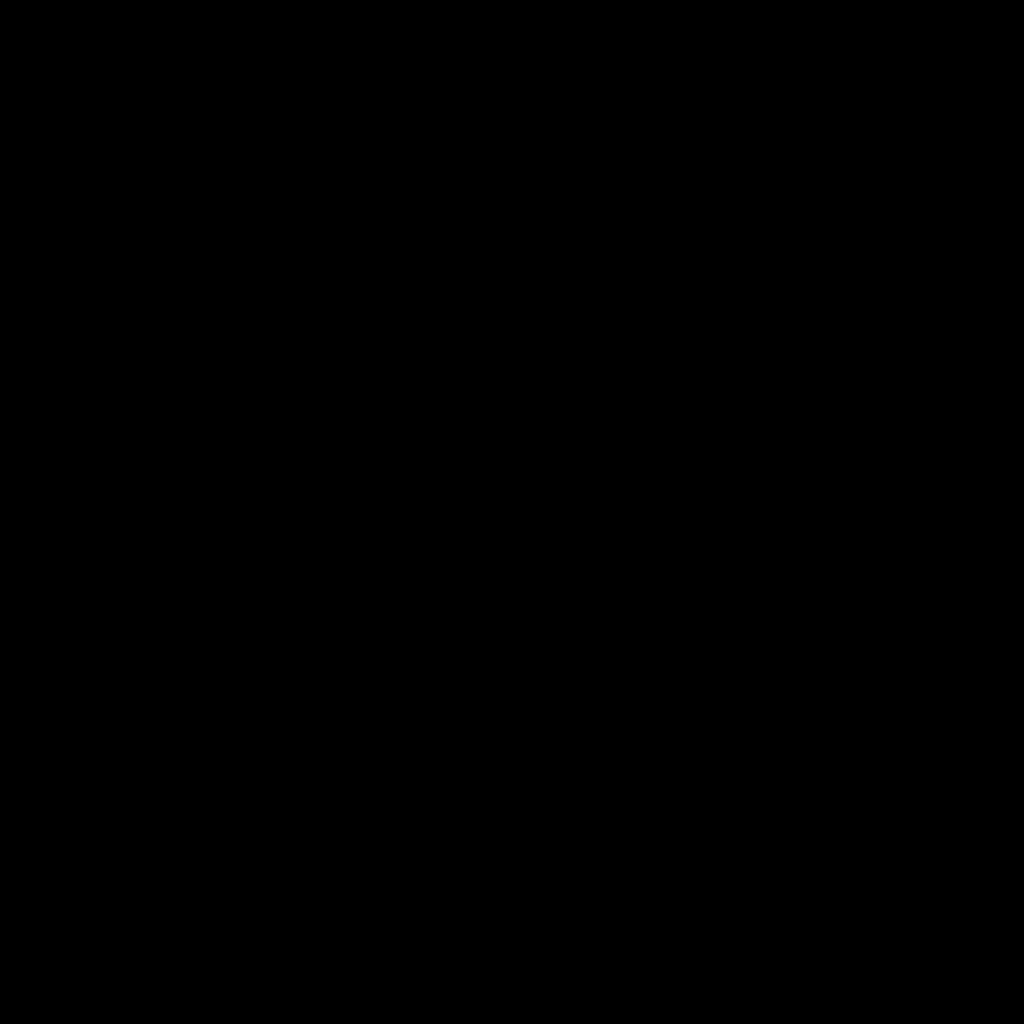

Supplement: Figure S6F Immunofluorescence of AcTub (green) and CFAP45 (red) in spermatozoa released from the caudal epididymis of Cfap52+/+ and Cfap52–/– mice. Nuclei were stained with DAPI (blue). [file mmc8.zip › Figure 6F/Image 56/Image 56_c2.tif]

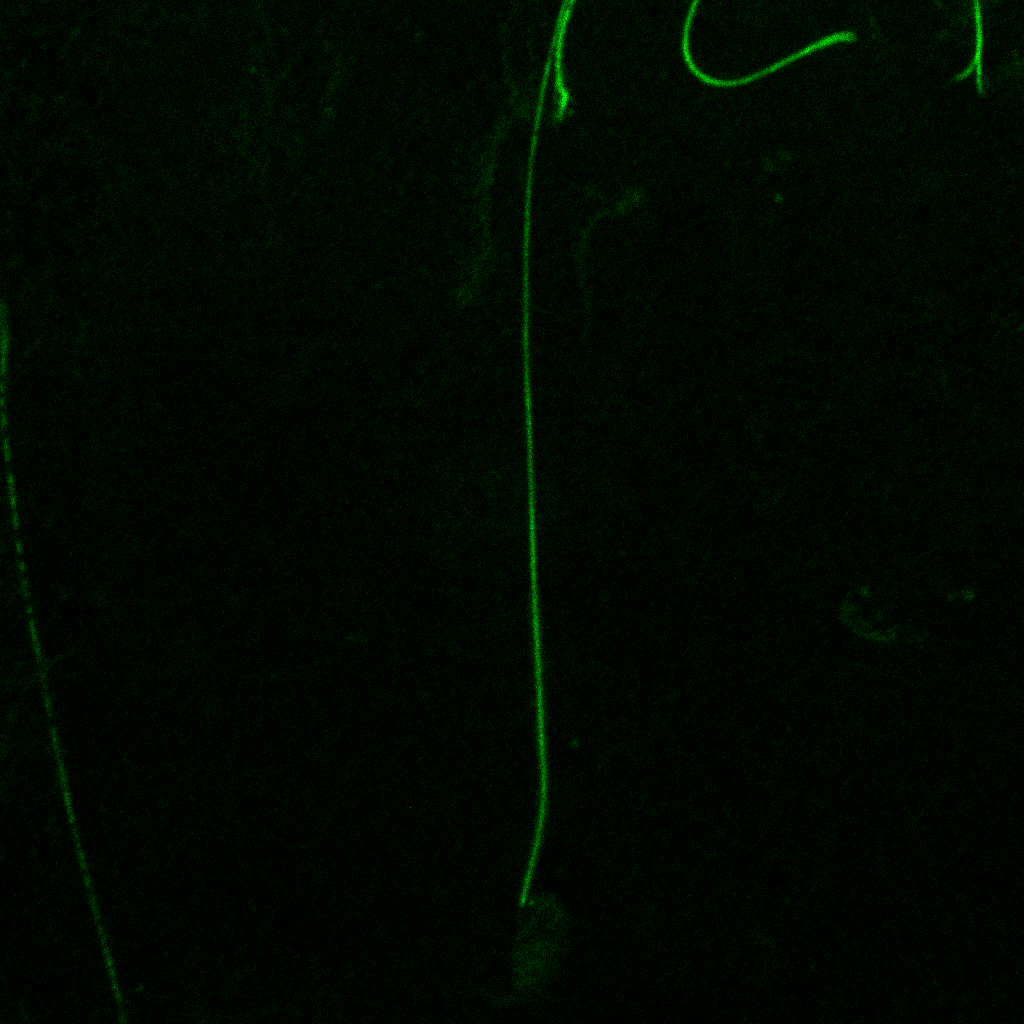

Supplement: Figure S6F Immunofluorescence of AcTub (green) and CFAP45 (red) in spermatozoa released from the caudal epididymis of Cfap52+/+ and Cfap52–/– mice. Nuclei were stained with DAPI (blue). [file mmc8.zip › Figure 6F/Image 56/Image 56_c3.tif]

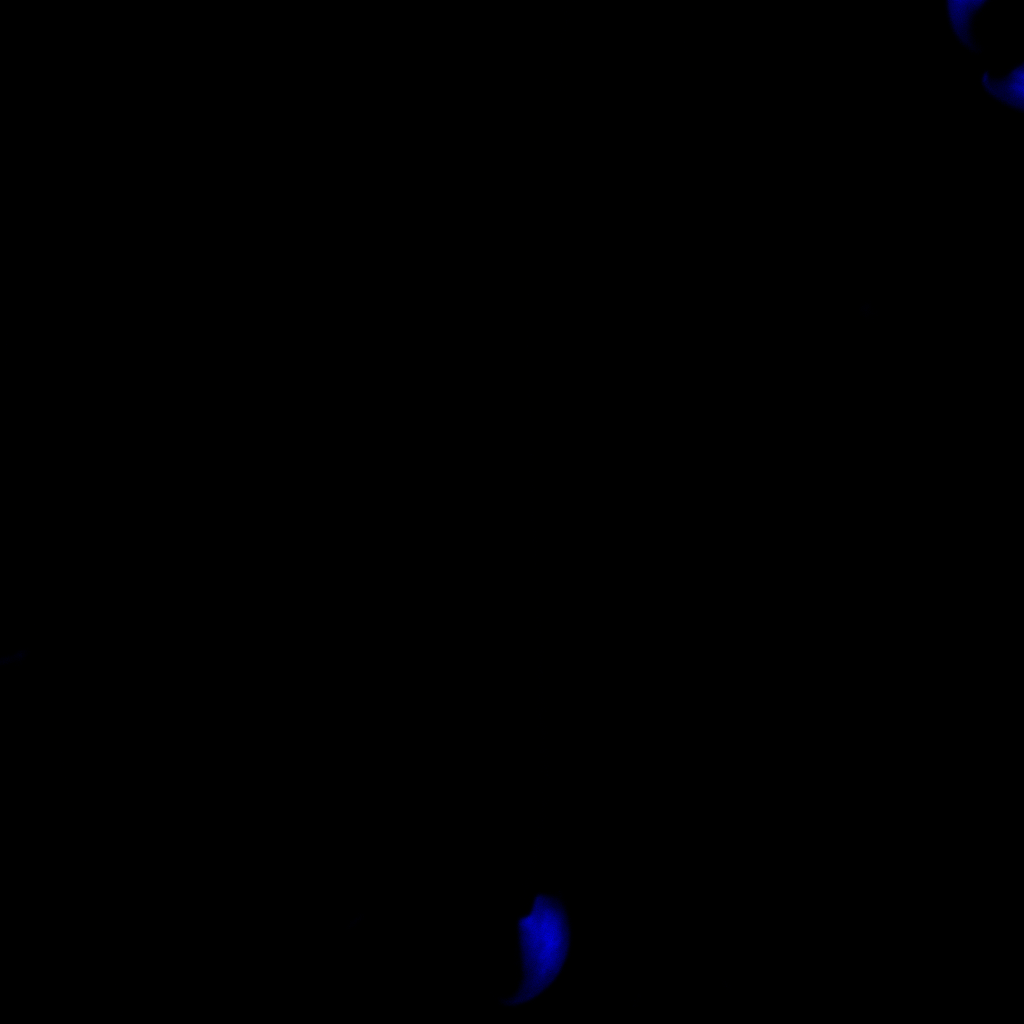

Supplement: Figure S6F Immunofluorescence of AcTub (green) and CFAP45 (red) in spermatozoa released from the caudal epididymis of Cfap52+/+ and Cfap52–/– mice. Nuclei were stained with DAPI (blue). [file mmc8.zip › Figure 6F/Image 56/Image 56_c4.tif]

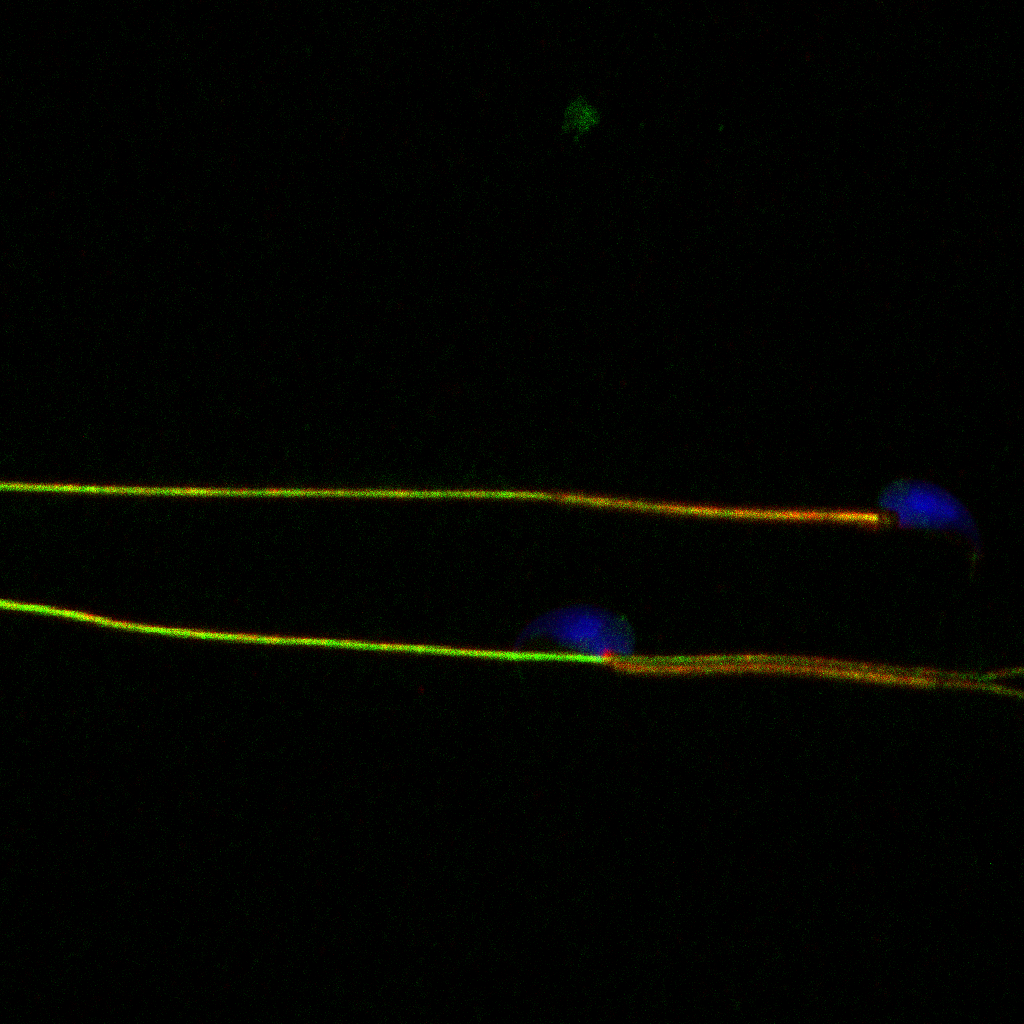

Supplement: Figure S6F Immunofluorescence of AcTub (green) and CFAP45 (red) in spermatozoa released from the caudal epididymis of Cfap52+/+ and Cfap52–/– mice. Nuclei were stained with DAPI (blue). [file mmc8.zip › Figure 6F/Image 62/Image 62_c1+2+3+4.tif]

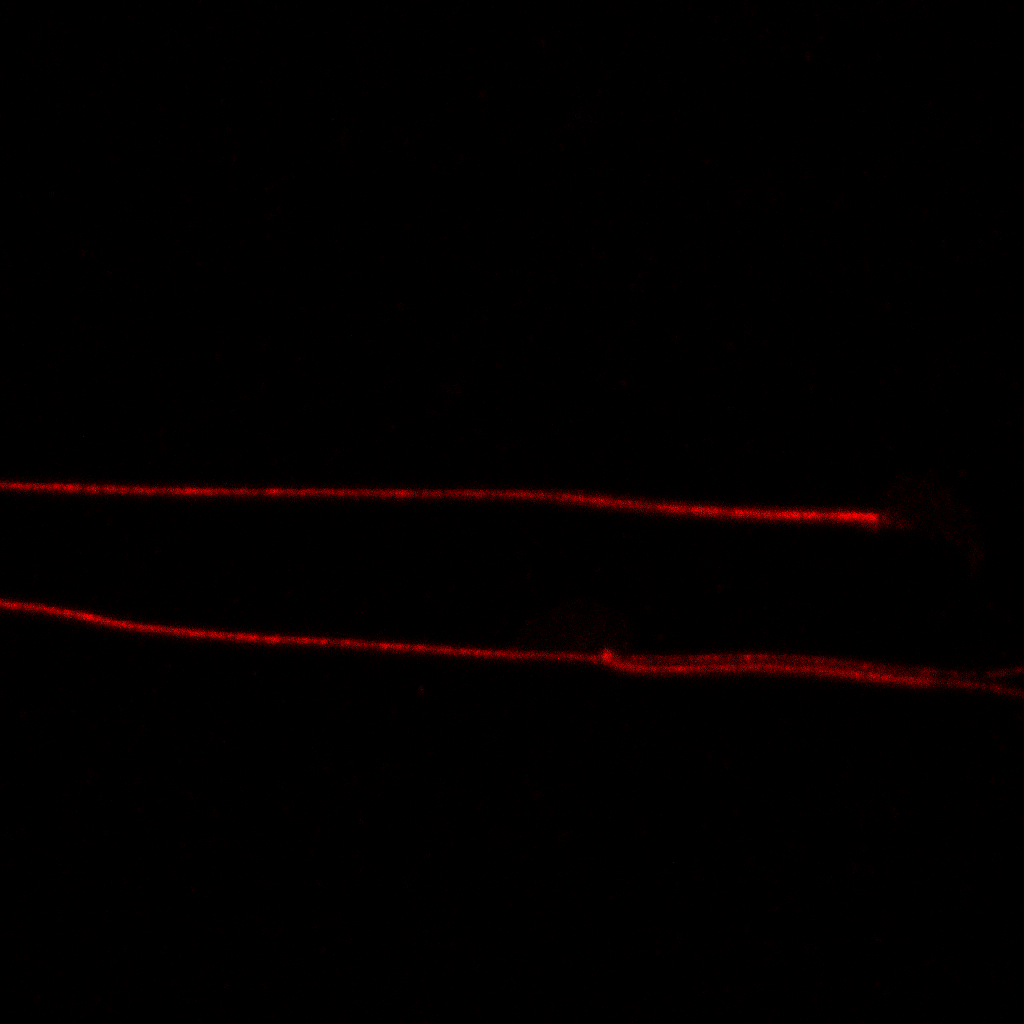

Supplement: Figure S6F Immunofluorescence of AcTub (green) and CFAP45 (red) in spermatozoa released from the caudal epididymis of Cfap52+/+ and Cfap52–/– mice. Nuclei were stained with DAPI (blue). [file mmc8.zip › Figure 6F/Image 62/Image 62_c1.tif]

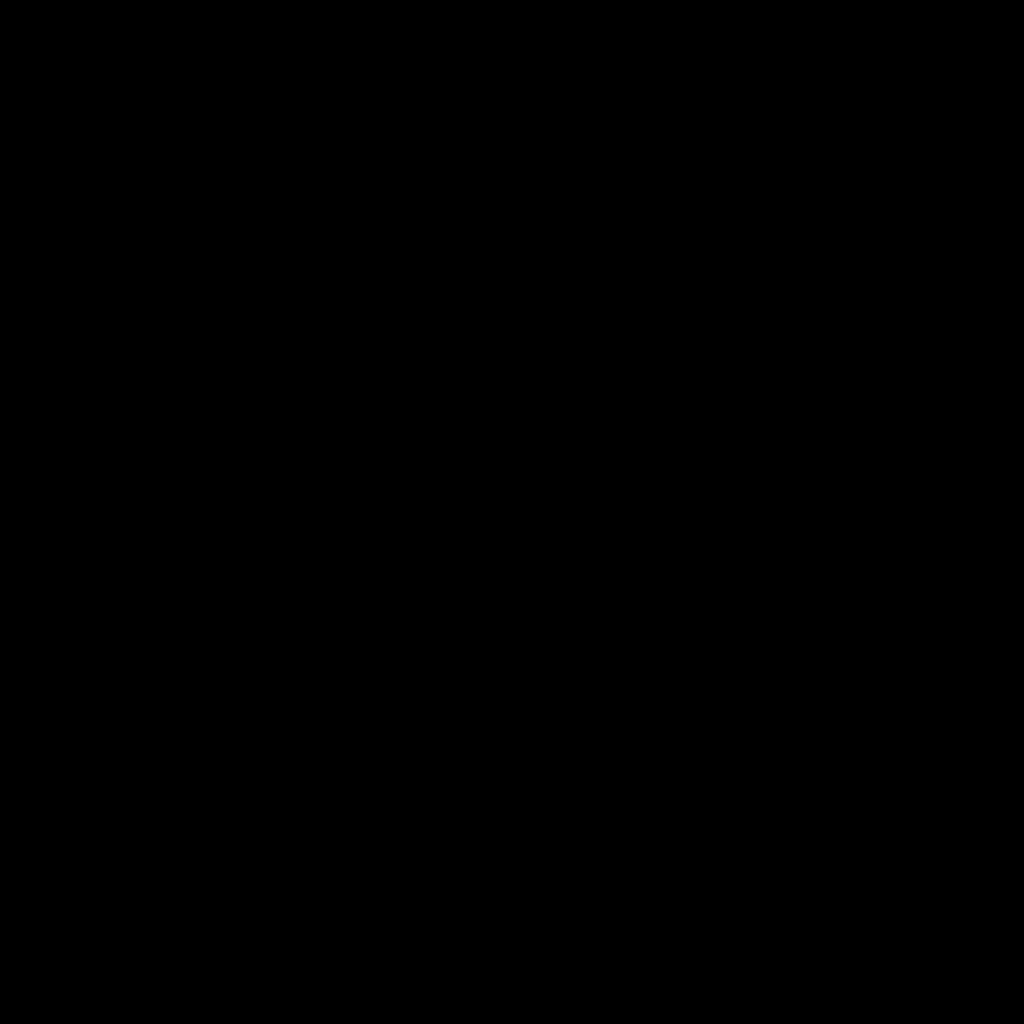

Supplement: Figure S6F Immunofluorescence of AcTub (green) and CFAP45 (red) in spermatozoa released from the caudal epididymis of Cfap52+/+ and Cfap52–/– mice. Nuclei were stained with DAPI (blue). [file mmc8.zip › Figure 6F/Image 62/Image 62_c2.tif]

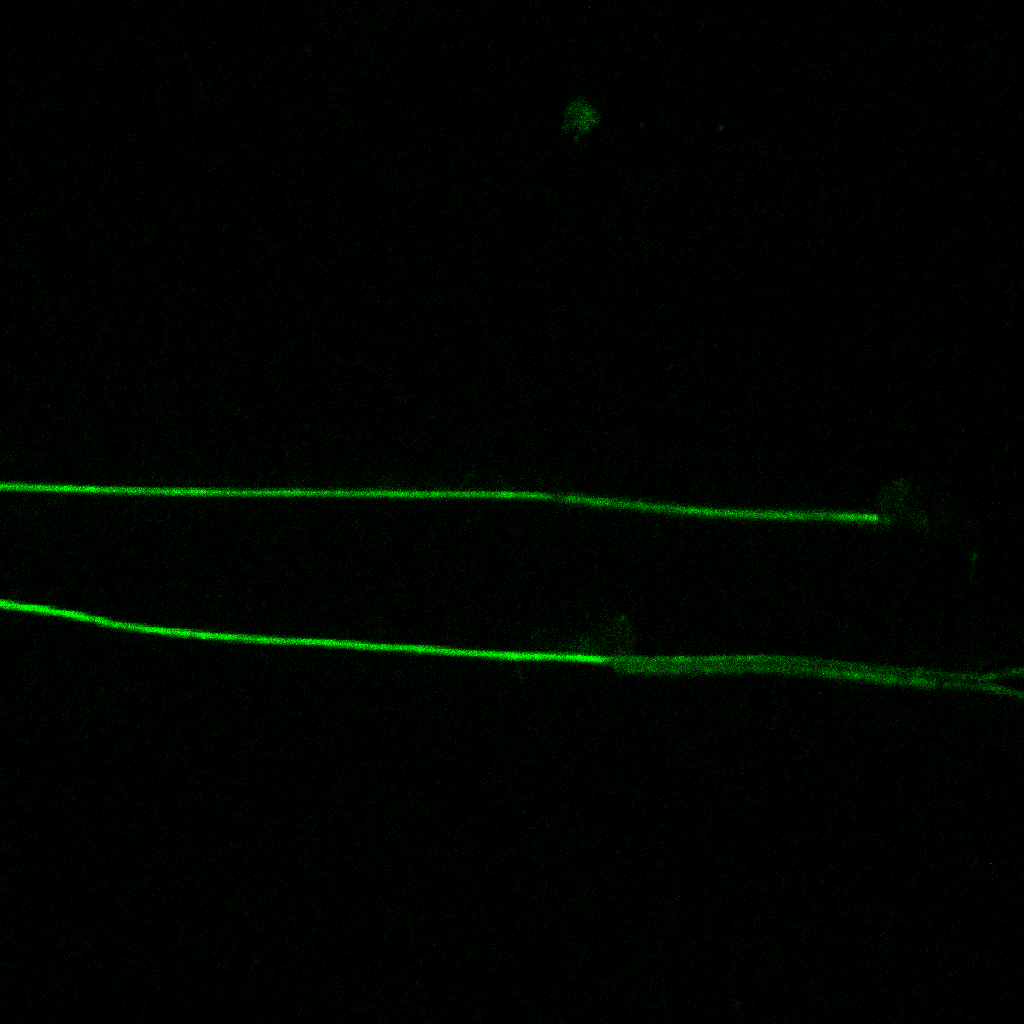

Supplement: Figure S6F Immunofluorescence of AcTub (green) and CFAP45 (red) in spermatozoa released from the caudal epididymis of Cfap52+/+ and Cfap52–/– mice. Nuclei were stained with DAPI (blue). [file mmc8.zip › Figure 6F/Image 62/Image 62_c3.tif]

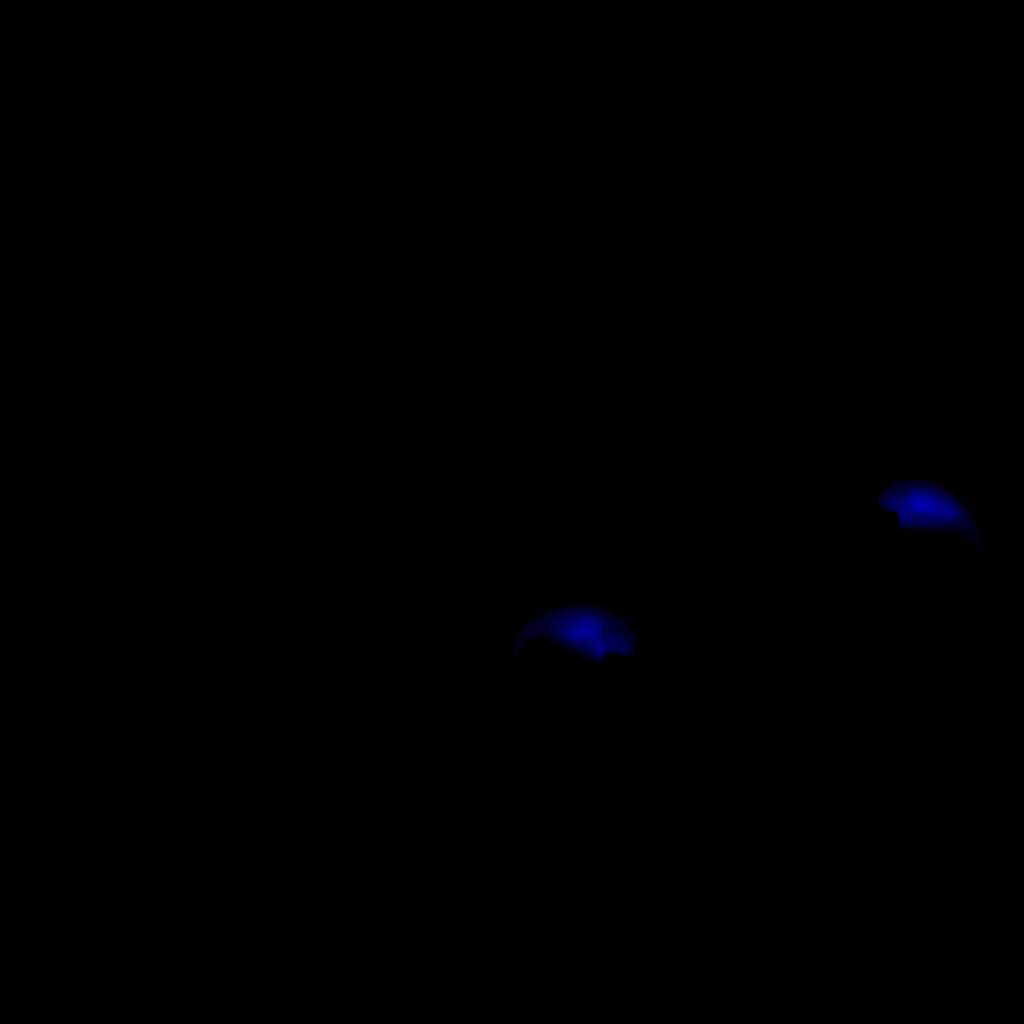

Supplement: Figure S6F Immunofluorescence of AcTub (green) and CFAP45 (red) in spermatozoa released from the caudal epididymis of Cfap52+/+ and Cfap52–/– mice. Nuclei were stained with DAPI (blue). [file mmc8.zip › Figure 6F/Image 62/Image 62_c4.tif]

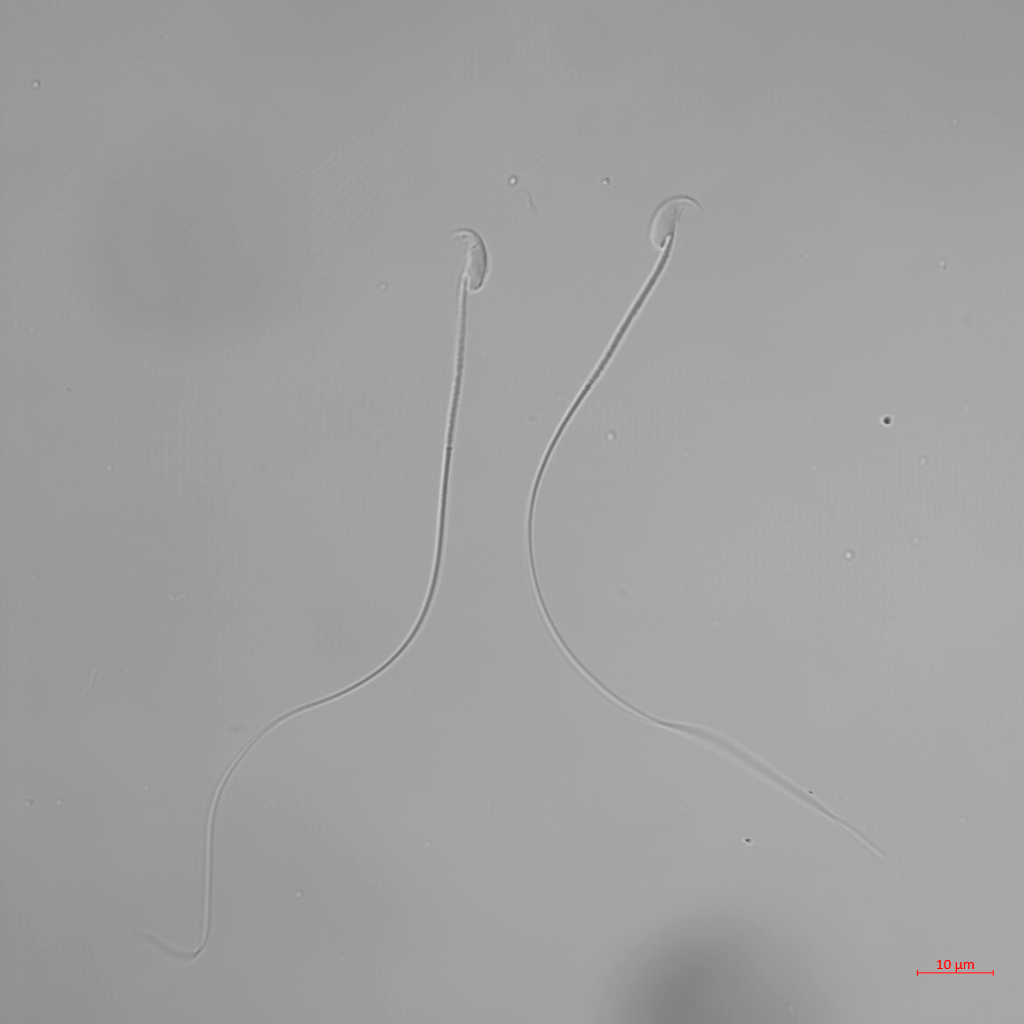

Supplement: Figure S6H Microtubule sliding assay to reconstitute dynein ATPase activity. Microtubule sliding was observed (black arrows). [file mmc9.zip › Figure 6H/Image 73_c1+2.tif]

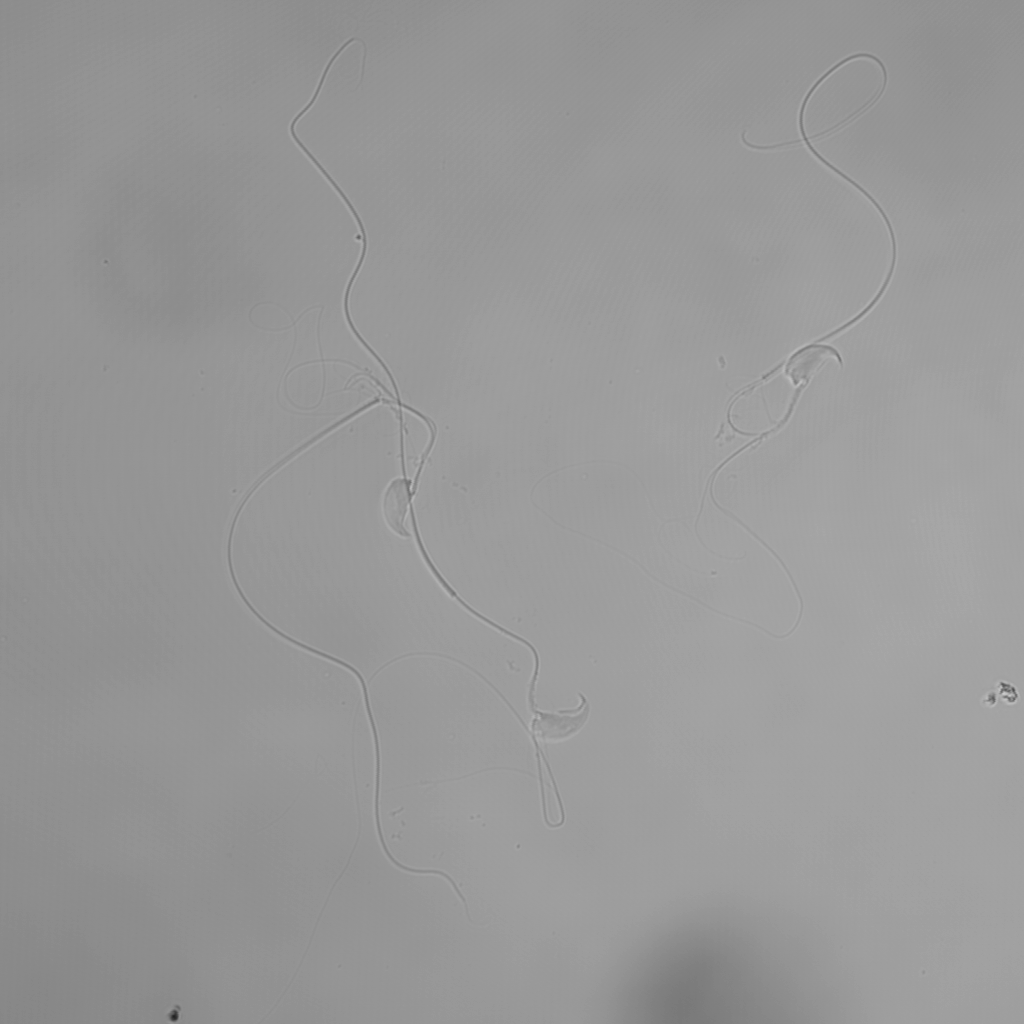

Supplement: Figure S6H Microtubule sliding assay to reconstitute dynein ATPase activity. Microtubule sliding was observed (black arrows). [file mmc9.zip › Figure 6H/OK_c1+2.tif]
